# Genetic parameters for first lactation dairy traits in the Alpine and Saanen goat breeds using a random regression test-day model

**DOI:** 10.1186/s12711-019-0485-3

**Published:** 2019-08-13

**Authors:** Mathieu Arnal, Hélène Larroque, Hélène Leclerc, Vincent Ducrocq, Christèle Robert-Granié

**Affiliations:** 1 GenPhySE, Université de Toulouse, INRA, ENVT, Toulouse INP, 31326 Castanet-Tolosan, France; 2 Institut de l’Elevage, Chemin de Borde Rouge, 31326 Castanet-Tolosan Cedex, France; 3 INRA UMR1313 GABI, Institut de l’Elevage, 78350 Jouy-en-Josas, France; 4 UMR1313 GABI, INRA, AgroParisTech, Université Paris-Saclay, 78350 Jouy-en-Josas, France

## Abstract

**Background:**

Random regression models (RRM) are widely used to analyze longitudinal data in genetic evaluation systems because they can better account for time-course changes in environmental effects and additive genetic values of animals by fitting the test-day (TD) specific effects. Our objective was to implement a random regression model for the evaluation of dairy production traits in French goats.

**Results:**

The data consisted of milk TD records from 30,186 and 32,256 first lactations of Saanen and Alpine goats. Milk yield, fat yield, protein yield, fat content and protein content were considered. Splines were used to model the environmental factors. The genetic and permanent environmental effects were modeled by the same Legendre polynomials. The goodness-of-fit and the genetic parameters derived from functions of the polynomials of orders 0 to 4 were tested. Results were also compared to those from a lactation model with total milk yield calculated over 250 days and to those of a multiple-trait model that considers performance in six periods throughout lactation as different traits. Genetic parameters were consistent between models. Models with fourth-order Legendre polynomials led to the best fit of the data. In order to reduce complexity, computing time, and interpretation, a rank reduction of the variance covariance matrix was performed using eigenvalue decomposition. With a reduction to rank 2, the first two principal components correctly summarized the genetic variability of milk yield level and persistency, with a correlation close to 0 between them.

**Conclusions:**

A random regression model was implemented in France to evaluate and select goats for yield traits and persistency, which are independent i.e. no genetic correlation between them, in first lactation.

**Electronic supplementary material:**

The online version of this article (10.1186/s12711-019-0485-3) contains supplementary material, which is available to authorized users.

## Background

Random regression test-day (TD) models (RRM) are widely used in genetic evaluations of TD milk production in dairy cows but also in other species such as dairy goats [1–6]. RRM increase the accuracy of breeding value predictions and fit the variability of environmental effects throughout lactation more accurately [7].

Besides describing the variability of genetic parameters along the lactation curve, RRM serve to predict estimated breeding values (EBV) for lactation persistency, based on the variation of EBV throughout lactation. A persistent animal is defined as producing on average less milk at the beginning but more at the end of the lactation period than animals with a similar overall production [8]. Lactation persistency is of interest for dairy producers because the shape of the lactation curve can affect an animal’s nutritional needs, and consequently its health, as well as the distribution of the farm’s milk output during the year [9, 10]. In a previous study, Arnal et al. [11] demonstrated the phenotypic variability of the shape of the dairy goat lactation curves in France, and showed that the main environmental factors influencing curve shapes are breed, kidding month, age at kidding, gestation stage, and length of the dry period.

Various functions have been used to model fixed, genetic and permanent environment effects on TD records, including the Wilmink function [12], Legendre polynomials [13, 14], and splines [15, 16]. Given that RRM are computationally expensive, Legendre polynomial functions have the computational advantage of reducing the correlations between estimated regression coefficients, which impact the convergence [7] of the iterative algorithms used for variance component estimation and genetic evaluation. They are also sufficiently flexible to fit differently shaped curves.

With eigen decomposition, it becomes possible to reduce the rank of the resulting variance–covariance matrix by ignoring the contribution of the smallest eigenvalues and eigenvectors [14, 17]. This decreases computing time by reducing the number of genetic and permanent environment regression coefficients that are estimated.

To date, the implementation of RRM in dairy goats has not been studied in France. In this paper, we estimate genetic parameters for milk yield, fat yield, protein yield, fat content, and protein content using RRM with Legendre polynomial functions of different orders, with or without rank reductions, to obtain TD EBV for French dairy goats in first lactation.

## Methods

### Data

The data consisted of 193,226 milk TD records from 30,186 first-lactation Saanen goats (234 herds) and 205,841 milk TD records from 32,256 first-lactation Alpine goats (198 herds) from northwestern France, collected between 1995 and 2015. The pedigree consisted of 66,716 and 67,159 Saanen and Alpine goats, respectively. Each lactation included at least four TD between the 7th and 270th day in milk (DIM). Lactation had to last between 180 and 350 days. Goats were milked twice a day and their records were summed to obtain their daily production. More than four animals per herd × test date combination were required. The sires of these goats were artificial-insemination bucks with at least 20 progeny in the dataset, with 379 and 324 sires from the Alpine and Saanen goat breeds, respectively. The dams of the goats had to be known. The traits analyzed were milk, fat and protein yields and fat and protein contents.

### Random regression models (RRM)

Five RRM were tested separately for each breed and for all five traits and all had the form:

$$y_{tijkldgn} = HTD_{ti} + {\text{A}}_{tjk} + {\text{M}}_{tjl} + \mathop \sum \limits_{r = 1}^{6} \theta_{tkr} N_{{\left( {r,d} \right)}} + \mathop \sum \limits_{r = 1}^{6} \tau_{tlr} N_{{\left( {r,d} \right)}} + \mathop \sum \limits_{s = 1}^{4} \gamma_{ts} M_{{\left( {s,g} \right)}} + \mathop \sum \limits_{o = 0}^{q} a_{tno} \varphi_{{\left( {o,d} \right)}} + \mathop \sum \limits_{o = 0}^{q} p_{tno} \varphi_{{\left( {o,d} \right)}} + e_{tijkldgn} ,$$

where $y_{tijkldgn}$ is the observation for trait $t$ (milk yield, fat yield, protein yield, fat content, protein content) on day in milk (DIM) $d$ (from 7 to 270 day) of goat $n$ that was pregnant for the past $g$ days ($g = 0$ if not pregnant), was in production year $j$ (from 1995 to 2015), belonged to kidding age class $k$ (7 classes [in months]: 9–11, 12, 13, 14, 15, 16, 17 or more), to kidding period class $l$ (8 classes: January, February, March, April–May–June, July–August–September, October, November, December), and to herd × test-date class $i$; $HTD_{ti}$ is a herd-test-date fixed effect; ${\text{A}}_{tjk}$ is the fixed effect of year-age at kidding; ${\text{M}}_{tjl}$ is the fixed effect of year-kidding period; $\theta_{tkr} , \tau_{tlr}$ and $\gamma_{ts}$ are the fixed regression coefficients for age at kidding, kidding period and gestation stage, respectively; $N_{{\left( {r,d} \right)}}$ is covariate $r$ at time $d$ of a cubic natural spline function with six knots at $d = 7, 20, 50, 110, 190, 270$; $M_{{\left( {s,g} \right)}}$ is covariate $s$ at time $d$ of a cubic natural spline function with four knots at $d$ = 31, 53,76, and 100 (at $g$ between 0 and 30 day, the coefficients were assumed to be equal to 0, implying that there was no effect of gestation on production; due to scarcity of records, the effect of gestation was considered to be constant after $g = 100$). $a_{tno}$ is the set of random additive genetic regression coefficients that follow a normal distribution $\left( {Var \left( {a_{to} } \right)\sim N\left( {0,\upsigma_{{a_{to} }}^{2} } \right)} \right)$ for the $o$th term of a Legendre polynomial of order $q$, $p_{tno}$ is the set of random permanent environmental regression coefficients that follow a normal distribution $\left( {Var\left( {p_{to} } \right)\sim N\left( {0,\upsigma_{{{\text{p}}_{\text{to}} }}^{2} } \right)} \right)$ for the $o$th term of a Legendre polynomial of order $q$, $\varphi_{{\left( {o,d} \right)}}$ is the value of the $o$th term of the Legendre polynomial at time $d$ and $e_{tijkldgn}$ is the normally-distributed residual term. The model assumed a heterogeneous residual variance along lactation that was considered to be constant within nine classes of DIM (7–36, 37–66, 67–96, 97–126, 127–156, 157–186, 187–216, 217–246, 247–270), without between-class correlations.

Legendre polynomials of order 0 to 4 (${\text{leg}}0$ to ${\text{leg}}4$) were tested assuming the same order for both the random genetic and environmental effects. The EBV of a goat for a complete lactation milk yield was obtained by summing the EBV for each DIM (called ${\text{SUM}}\_{\text{leg}}q$) [18]. Given that the RRM can give an estimation of the shape of the lactation curve for each goat, EBV for persistency (denoted ${\text{PERS}}\_{\text{leg}}q$) was computed as the cumulative deviation in genetic contribution to yield from the DIM 40 to DIM 240 relative to an average animal having the same yield at DIM 40 [18].

The next step was to test various rank reductions of the genetic variance–covariance matrices based on an eigen decomposition of these matrices. To keep a consistent definition of persistency, the second eigenvector was set, by multiplying by − 1, to be systematically negative at the beginning of lactation, and then positive. For a reduction to rank $z$, the first $z$ eigenvectors obtained with the complete model were multiplied by the terms of Legendre polynomial as in Druet et al. [14] to obtain the $z$ first eigenfunctions. These eigenfunctions were used to model the genetic and permanent environment effects. The Legendre polynomial function of order $q$ with a covariance matrix reduced (RRM*) to rank $z$ is written ${\text{leg}}q{\text{R}}z$. The reduced models were of the form:

$$y_{tijkldgn} = HTD_{ti} + {\text{A}}_{tjk} + {\text{M}}_{tjl} + \mathop \sum \limits_{r = 1}^{6} \theta_{tkr} N_{{\left( {r,d} \right)}} + \mathop \sum \limits_{r = 1}^{6} \tau_{tlr} N_{{\left( {r,d} \right)}} + \mathop \sum \limits_{s = 1}^{4} \gamma_{ts} M_{{\left( {s,g} \right)}} + \mathop \sum \limits_{o = 1}^{z} b_{tno} \chi_{{\left( {o,d} \right)}} + \mathop \sum \limits_{o = 1}^{z} c_{tno} \chi_{{\left( {o,d} \right)}} + e_{tijkldgn} ,$$

where $b_{tno}$ is the random additive genetic regression coefficient that follows a normal distribution $\left( {Var\left( {b_{to} } \right)\sim N\left( {0,\upsigma_{{{\text{b}}_{\text{to}} }}^{2} } \right)} \right)$ for the $o$th eigenfunction of the genetic (co)variances matrix obtained with a $q$th-order Legendre polynomial model reduced to rank $z$, $c_{tno}$ is the random permanent environmental regression coefficient that follows a normal distribution $\left( {Var\left( {c_{to} } \right)\sim N\left( {0,\upsigma_{{{\text{c}}_{\text{to}} }}^{2} } \right)} \right)$ for the $o$th eigenfunction of the genetic (co)variances matrix obtained with a $q$th-order Legendre polynomial model reduced to rank $z$, $\chi_{{\left( {o,d} \right)}}$ is the value of the $o$th eigenfunction of the genetic (co)variances matrix obtained with a $q$th-order Legendre polynomial model reduced to rank $z$ ($z = \left\{ {2,3} \right\}$) at DIM $d$.

The corresponding EBV for whole lactation performance of goat $n$ from the RRM was obtained by summing its EBV throughout the DIM period (from DIM 7 to DIM 270) (${\text{SUM}}\_{\text{legxRz}}$).

#### Multiple-trait model (MT)

The evolution of the estimated heritabilities throughout the DIM period and the genetic correlations between DIM that were obtained with RRM were compared to those obtained with a MT model, in which lactation was subdivided into six periods (1 = [7, 45], 2 = [46, 90], 3 = [91, 135], 4 = [136, 180], 5 = [181, 225], 6 = [226, 270]). TD yields and contents for each period $r$ ($r$= 1 to 6) were considered as different traits. The frequency of TD recording (on average every 28 or 35 days) implied that only one TD record was performed normally per period but if two TD were carried out, the measures were averaged. The MT model was:

$$y_{tijklnro} = \pi_{tir} + \delta_{tjr} + \gamma_{tkr} + HY_{tlr} + a_{tnr} + e_{tijklnro} ,$$

where $y_{tijklnro}$ is the observation for trait $t$ (milk yield, fat yield, protein yield, fat content, protein content) of goat $n$ for period $r$ ($r$ =1 to 6), in subclass $i$ for $\pi$, the age at kidding effect, subclass $j$ for $\delta$, the month at kidding effect, subclass $k$ for $\gamma$, the gestation stage effect at DIM 270 (the last DIM studied), and class $l$ for the herd-year (HY) effect. The fixed effects classes are the same as in the previous RRM. $a_{nr}$ is the additive genetic breeding value of animal $n$ for period $r$ and followed a normal distribution $\left( {Var\left( {a_{r} } \right)\sim N\left( {0,\upsigma_{{{\text{MT}}_{\text{r}} }}^{2} } \right)} \right)$.

#### Lactation model (LACT)

We implemented a lactation (LACT) model that was close to that routinely used for official genetic evaluations in France. The total milk, fat and protein yields throughout lactation were calculated as in routine genetic evaluations using the Fleischmann method [19]. Fat and protein contents were derived from the total lactation yields. The genetic evaluation model based on these phenotypes was:

$$y_{ijkln} = \pi_{i} + \delta_{j} + \gamma_{k} + HY_{l} + a_{n} + e_{ijklm} ,$$

where $y_{ijklm}$ was the lactation observation for trait $t$ (milk yield, fat yield, protein yield, fat content, protein content) of goat $n$, in subclass $i$ for $\pi$, the age at kidding effect, subclass $j$ for $\delta$, the month at kidding effect, subclass $k$ for $\gamma$, the gestation stage effect at DIM 270 (the last DIM studied), and class $l$ for herd-year effect ($HY$). Fixed effects were defined as in the previous RRM. $a_{n}$ was the additive genetic breeding value of animal $n$ and followed a normal distribution (mean: $\upmu_{\text{LACT}} = 0$, variance is $\upsigma_{\text{LACT}}^{2}$).

All the genetic parameters were estimated using the WOMBAT software [20].

#### Estimation of genetic correlations and heritabilities with the RRM

For each trait, the heritability of the $o$th regression coefficients (${\text{h}}\_{\text{b}}$) was calculated as in Schaeffer [21], by dividing the genetic variance of the $o$th regression coefficient by the sum of the genetic variance of the $o$th regression coefficient, the permanent environment variance of the $o$th regression coefficient, and the mean square error.

For each trait, the genetic variance–covariance matrix between all DIM was obtained following Druet et al. [14] as ${\mathbf{G}}_{264} = {\mathbf{QK}}_{g} {\mathbf{Q^{\prime}}}$, where ${\mathbf{G}}_{264}$ is a 264-by-264 genetic variance–covariance matrix, ${\mathbf{Q}}$ is a 264-by-$q$ matrix with the (daily) values of the $q$ terms of the Legendre polynomial, and ${\mathbf{K}}_{g}$ is the $q$-by-$q$ genetic variance–covariance matrix. The same approach was used to obtain the permanent environmental variance–covariance matrix ${\mathbf{W}}_{264}$. The phenotypic variance–covariance matrix between all DIM, ${\mathbf{P}}_{264}$, was obtained by summing ${\mathbf{G}}_{264}$, ${\mathbf{W}}_{264}$ and the residual variance for the relevant DIM.

Heritabilities for each test day were obtained by dividing the diagonal elements of ${\mathbf{G}}_{264}$ by the corresponding diagonal elements of ${\mathbf{P}}_{264}$.

The genetic correlations between DIM $d$ and the other DIM were derived from ${\mathbf{G}}_{264}$.

Genetic variances for each trait throughout the whole lactation ($$g_{wl} )$$ were obtained following Hammami et al. [22] as $$g_{wl} = {\mathbf{s}} {\mathbf{G}}_{264} {\mathbf{s^{\prime}}}$$, where $${\mathbf{s}}$$ is a summation vector (vector of 1 s) of length 264. The same approach was used to obtain the permanent environmental ($$w_{wl} )$$ and phenotypic variances for the whole lactation $$p_{wl}$$. The heritability of each trait on the lactation scale ($${\text{h}}\_{\text{wl}}$$) was obtained by dividing $$g_{wl}$$ by $$p_{wl}$$.

#### Criteria for comparing the models

The goodness-of-fit of the models for each trait was assessed by comparing the Bayesian information criterion (BIC) and the Pearson correlation coefficients ($\uprho$) between observed and predicted phenotypes for each model. Pearson correlation coefficients were also used to compare the EBV of the bucks obtained from different models.

## Results

### Rank reduction of the variance–covariance matrix of the most complex model

In the Eigen decomposition of the genetic matrix from the ${\text{leg}}4$ model, the first two principal components (PC) represented on average more than 97% of the total genetic variance (88 and 9%, respectively), and Additional file 1: Table S1 shows the percentage of variance represented by each PC for the five traits and the two breeds. The proportion of variance accounted for by the first eigenvalue was higher for yield traits and fat content in Saanen than in Alpine goats (from + 2% for fat content to + 4.5% for fat yield). In contrast, the proportion of variance accounted for by the third eigenvalue was higher in Alpine than in Saanen goats, although for all the traits it represented a very small fraction of the total variance (less than 4.2%).

The first eigenfunction was almost constant throughout the DIM period, which suggests that the first PC can be regarded as linked with the average production level throughout lactation, whereas the second PC varied almost linearly, which indicates extreme production levels at the beginning and end of lactation independently of the average production level, and thus it is associated with persistency. The correlations between these measures of average production level throughout lactation and persistency are equal to 0 by construction. The third eigenfunction showed contrasted production characteristics between those measured in the middle of lactation and those measured at the beginning and end of lactation. We found no significant between-trait differences in the shapes of eigenfunctions, see Additional file 2: Figure S1 that shows the eigenfunctions of each PC for the five traits in the Saanen breed.

### Model fitting

Table 1 shows the BIC values for each trait and both breeds with the five complete models (${\text{leg}}0$ to ${\text{leg}}4$) and the five reduced models (${\text{leg}}x{\text{R}}z$ with $x = \left\{ {2, 3, 4} \right\}$ and $z = \left\{ {2,3} \right\}$).

**Table 1:** Bayesian information criterion^a^ (BIC) for the five complete and five reduced models

	Saanen					Alpine				
	Milk yield	Fat yield	Protein yield	Fat content	Protein content	Milk yield	Fat yield	Protein yield	Fat content	Protein content
Complete model										
${\text{leg}}0$	19165	11543	13089	4229	14490	21314	14748	13014	4921	18671
${\text{leg}}1$	7008	2631	4253	1749	4555	6539	2578	3392	1648	4913
${\text{leg}}2$	1472	525	1023	747	1773	1739	835	975	826	2113
${\text{leg}}3$	293	81	201	231	788	293	197	220	285	947
${\text{leg}}4$	0	0	0	0	0	0	0	0	0	0
Reduced model										
${\text{leg}}2{\text{r}}2$	3962	1967	3382	1737	4852	4575	2617	3259	1647	4769
${\text{leg}}3{\text{r}}2$	3715	2258	3439	1825	4889	3827	2843	3352	1668	4858
${\text{leg}}4{\text{r}}2$	3699	2307	3442	1765	4931	3794	2822	3292	1632	4849
${\text{leg}}3{\text{r}}3$	1285	499	936	438	1521	952	612	906	570	1748
${\text{leg}}4{\text{r}}3$	1040	500	915	251	1426	873	619	895	481	1898

^a^Values are expressed as deviations from the best (smallest) value (${\text{leg}}4$)

Regardless of trait and breed, BIC decreased rapidly as the order of the Legendre polynomials increased from 0 to 2. The decay was smaller between orders 2 and 4 although 18 additional parameters had to be estimated. However, the differences between all models were significant (difference in BIC > 10), which indicates that, for all traits and both breeds, the fit to the data improved as the order of the Legendre polynomial used increased. As rank decreased, BIC decreased when the first three PC were kept instead of just the first two, without significant differences between ${\text{leg}}3{\text{R}}3$ and ${\text{leg}}4{\text{R}}3$. Regardless of the order of the Legendre polynomial, the BIC obtained by using two eigenfunctions of the genetic (co)variances matrix with model ${\text{leg}}x{\text{R}}2$ were close and often better (i.e. smaller) than with model ${\text{leg}}1$, with the same number of estimated parameters. The BIC obtained by using three eigenfunctions (${\text{leg}}x{\text{R}}3$) was similar and better than that with ${\text{leg}}2$, again with the same number of estimated parameters.

Pearson correlation coefficients (ρ) between observed data and predicted values were calculated to compare adjustment to data between traits and breeds and between RRM and MT models; Additional file 3: Table S2 presents the evolution of these correlations under the different models, for each trait in both breeds. For RRM, the conclusions drawn were similar to those for BIC. For the fourth-order Legendre polynomial, $\uprho$ were high for most traits (~ 0.96), but slightly lower for fat content in both breeds (0.93), which highlights a less satisfactory modeling of this trait than for the others. The MT model was the worst model for all traits, with $\uprho$ values ranging from 0.80 to 0.90, which can be explained by the genetic effect and certain fixed effects being constant throughout each period. With random regression models other than ${\text{leg}}0$, these effects gradually chance with DIM.

**Fig. 1:**
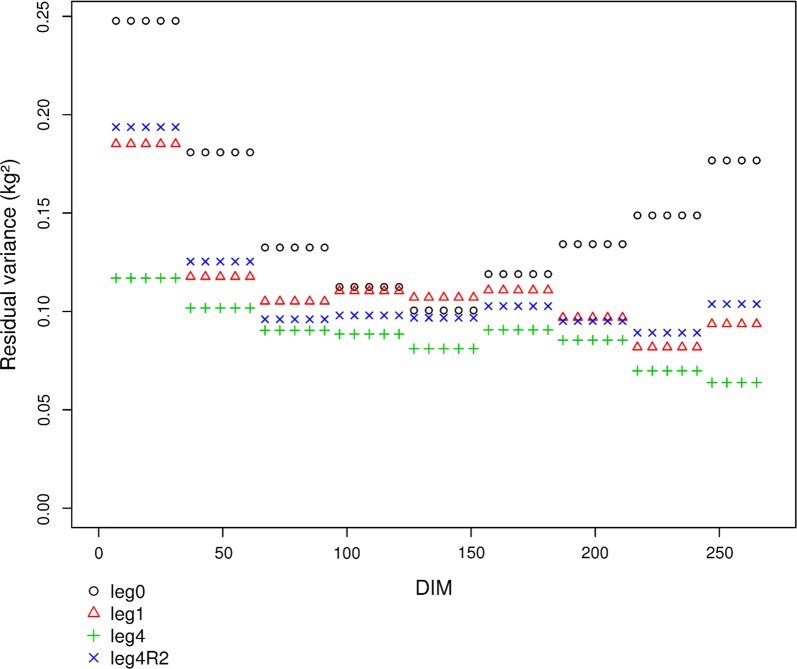
Evolution of residual variances with DIM for milk yield in Alpine goats

The evolution of the residual variance with DIM is another criterion for characterizing the effect of this variation on the quality of the adjustments throughout lactation. In the French dairy goat breeding program, the most important trait for cheese production is protein yield, which can also be expressed as protein content with respect to the level of milk yield. The residual variances for milk yield and protein content for different lactation periods are shown in Fig. 1 and Additional file 4: Figure S2, respectively. To keep the figures easy to interpret, the results for the ${\text{leg}}2$ and ${\text{leg}}3$ models are not presented, but these were very close to those of the ${\text{leg}}4$ model. The between-model differences in residual variance are larger at the beginning and end of lactation. As for BIC and $\uprho$, the higher is the order of the polynomial, the better is the adjustment, as indicated by the smaller residual variances. Figure 1 and Additional file 4: Figure S2 illustrate the better adjustment obtained with ${\text{leg}}4$ compared to the other models, at both ends of lactation. ${\text{leg}}x{\text{R}}3$ resulted in smaller residual variances than ${\text{leg}}x{\text{R}}2$, particularly at the beginning (DIM 7–45) and end of lactation (DIM 225–270) (results not shown). For clarity, the residuals of the reduced models derived from ${\text{leg}}2$ and ${\text{leg}}3$ were not included in the figures but were close to those from ${\text{leg}}4$. Overall, the RRM* adjustments were systematically much better than for the model that assumes a constant genetic value throughout lactation (${\text{leg}}0$). For model fit, similar results were found for the other traits in both breeds.

### Choice of final models

Model ${\text{leg}}4$ was chosen as the reference model for RRM because it resulted in a better fit to the data, but it is also the most complicated model in terms of number of genetic parameters to estimate. However, ${\text{leg}}0$ and ${\text{leg}}1$ appear too simplistic. In order to choose a robust model that is sufficiently manageable for large-scale routine evaluations (after extension to all lactations), we chose the ${\text{leg}}4{\text{R}}2$ model as an attractive compromise, since it derived from the best RRM and its interpretation was straightforward, i.e. it summarized two important lactation characteristics (average level and persistency). The third PC was considered less relevant because it accounted for much less variance. These different RRM (${\text{leg}}0$, ${\text{leg}}1$, ${\text{leg}}4{\text{R}}2$ and ${\text{leg}}4$) were compared to a lactation model (LACT) and a MT model for the estimation of genetic parameters.

### Heritability estimates

Heritabilities for each trait on the whole lactation (${\text{h}}\_{\text{wl}}$) scale were estimated as in Hammami et al. [22] with the LACT and different RRM models and were close to 0.3 for milk yield in both breeds and for fat and protein yields in Alpine goats, and slightly higher i.e. 0.34 for protein yield and 0.37 for fat yield in Saanen goats (see Additional file 5: Table S3). Heritability estimates reached 0.68 for fat content and 0.65 for protein content in Saanen, and 0.75 for fat content and 0.70 for protein content in Alpine goats. We observed no differences in heritability between the RRM for any of the traits. Furthermore, heritabilities estimated with the RRM* were close to those estimated with RRM. For all the traits, heritabilities estimated with the RRM were slightly higher, especially for fat content, than those estimated with the LACT model (0.65 for LACT and 0.75 with ${\text{leg}}4$ for fat content in the Alpine breed).

The heritabilities for the regression coefficients (${\text{h}}\_{\text{b}}$) estimated with the ${\text{leg}}4{\text{R}}2$ model as in Schaeffer [21] are in Table 2. These can be interpreted as heritability of average yield (PC1) and persistency of yield (PC2), respectively. The heritabilities for persistency (PC2) were close to 0.18 for protein content and ~ 0.10 for the other traits.

**Table 2:** Heritabilities of the regression coefficients (${\text{h}}\_{\text{b}}$) according to the PC derived from the ${\text{leg}}4{\text{R}}2$ model

	Saanen					Alpine				
	Milk yield	Fat yield	Protein yield	Fat content	Protein content	Milk yield	Fat yield	Protein yield	Fat content	Protein content
PC1	0.28	0.31	0.29	0.50	0.56	0.27	0.24	0.25	0.54	0.62
PC2	0.13	0.12	0.11	0.07	0.18	0.14	0.10	0.09	0.10	0.19

**Fig. 2:**
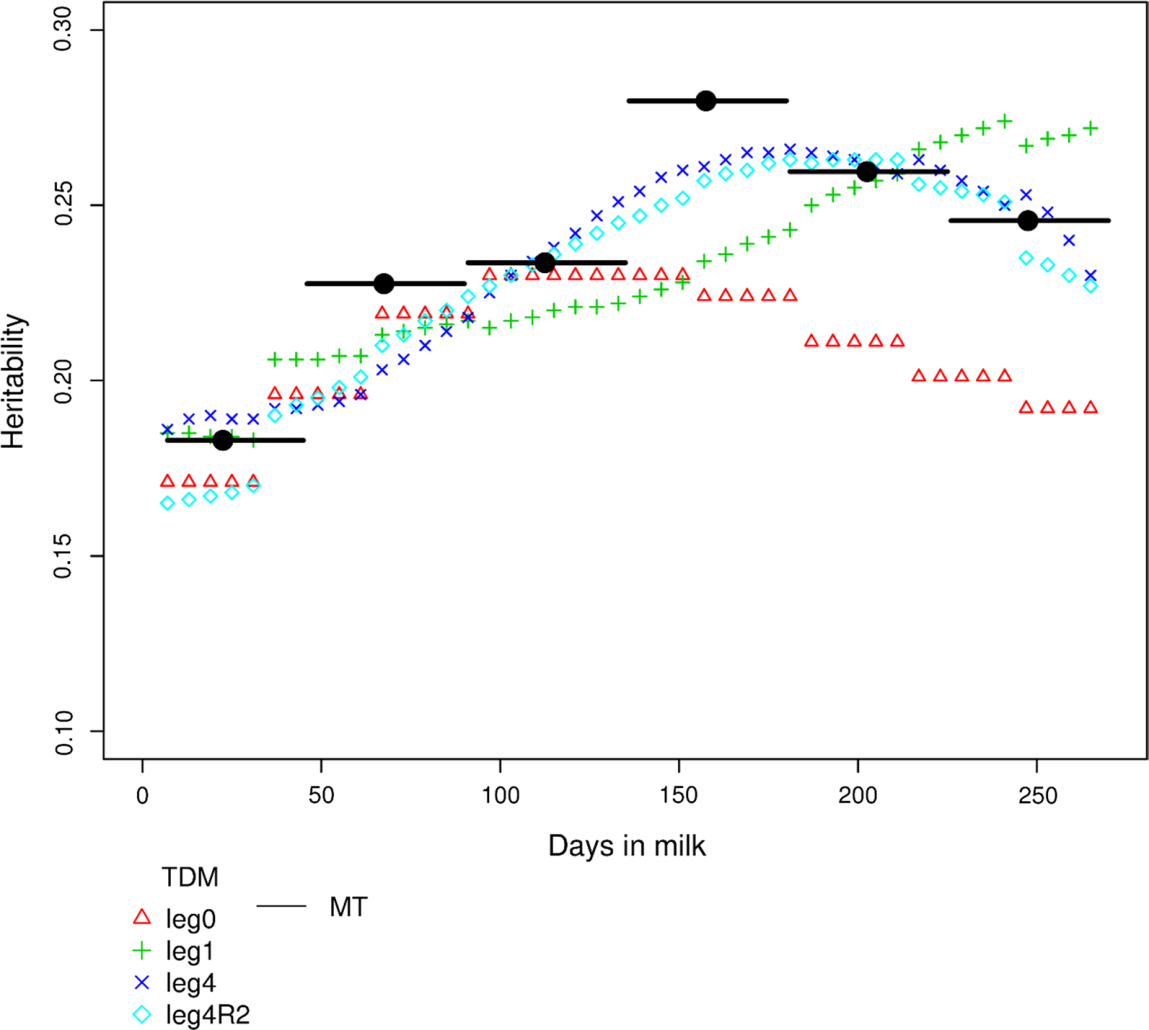
Estimated daily heritabilities for milk yield in Saanen goats

Figure 2 shows the evolution of the estimated heritabilities of test day yield throughout the lactation period obtained with the MT model and different RRM models for milk yield in the Saanen breed, i.e. ${\text{leg}}4$ and ${\text{leg}}4{\text{R}}2$, which were the models without and with reduction, ${\text{leg}}1$, which was a model that had the same number of parameters as ${\text{leg}}4{\text{R}}2$, and finally ${\text{leg}}0$. Models ${\text{leg}}4$ and ${\text{leg}}4{\text{R}}2$ yielded the highest heritabilities. The discontinuous curve of the estimated heritability throughout the DIM period is due to the residual variance that was different for each of six 30-day periods in the different RRM. The heritability estimated with the MT model first increased up to a peak in mid-lactation (0.26) and then decreased. However, ${\text{leg}}0$ yielded a lower heritability after DIM 150 for milk yield (due to a larger residual variance), whereas ${\text{leg}}1$ resulted in a different pattern with low heritabilities at the beginning but high heritabilities at the end of lactation. Additional file 6: Figure S3 shows the evolution of the estimated heritabilities throughout the DIM period for protein content in Saanen goats. The comparison between models resulted in similar results for milk yield and protein content, except with ${\text{leg}}1$ for protein content, which showed a profile closer to that of ${\text{leg}}4$. Similar results were also observed for the Alpine breed and the other traits. Estimated heritabilities obtained with RRM* were close to those calculated with MT and ${\text{leg}}4$ models, because their eigenfunctions are neither constant nor linear throughout lactation, unlike those of the ${\text{leg}}0$ and ${\text{leg}}1$ models.

**Fig. 3:**
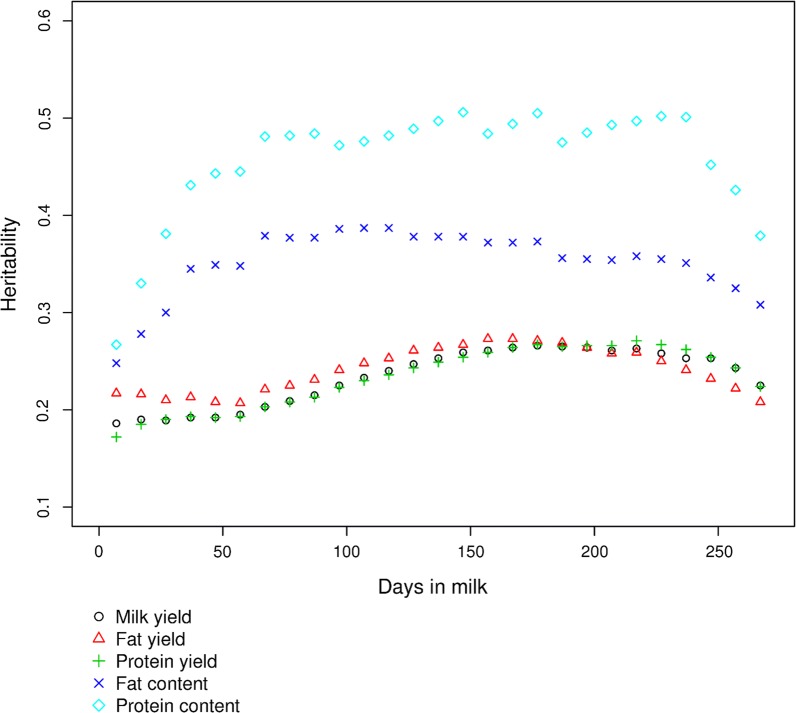
Evolution of heritabilities with DIM in Saanen goats with the ${\text{leg}}4$ model

Figure 3 for the Saanen breed and Additional file 7: Figure S4 for the Alpine breed present the heritabilities estimates for test days with the ${\text{leg}}4$ model throughout the lactation period for all five traits and show the same within-breed patterns for the three yield traits. The highest heritability estimated for milk yield was 0.26 in both breeds, and was reached on ~ DIM 175 and ~ DIM 125 for the Saanen and Alpine breeds, respectively. For component yield traits in the Alpine breed, the highest heritability of 0.23 was reached on ~ DIM 125, similar to that for milk yield. For component yield traits in the Saanen breed, the estimated heritabilities were highest, i.e. 0.27 on ~ DIM 157 for fat yield and ~ DIM 211 for protein yield. For protein and fat contents, the evolution of the heritabilities throughout the DIM period followed the same pattern in both breeds but values were higher in the Alpine breed i.e. 0.45 and 0.57, respectively for fat and protein content on ~ DIM 150 versus 0.38 for fat content on ~ DIM 106 and 0.51 for protein content on ~ DIM 150 in the Saanen breed.

### Genetic correlations between DIM from different models

**Fig. 4:**
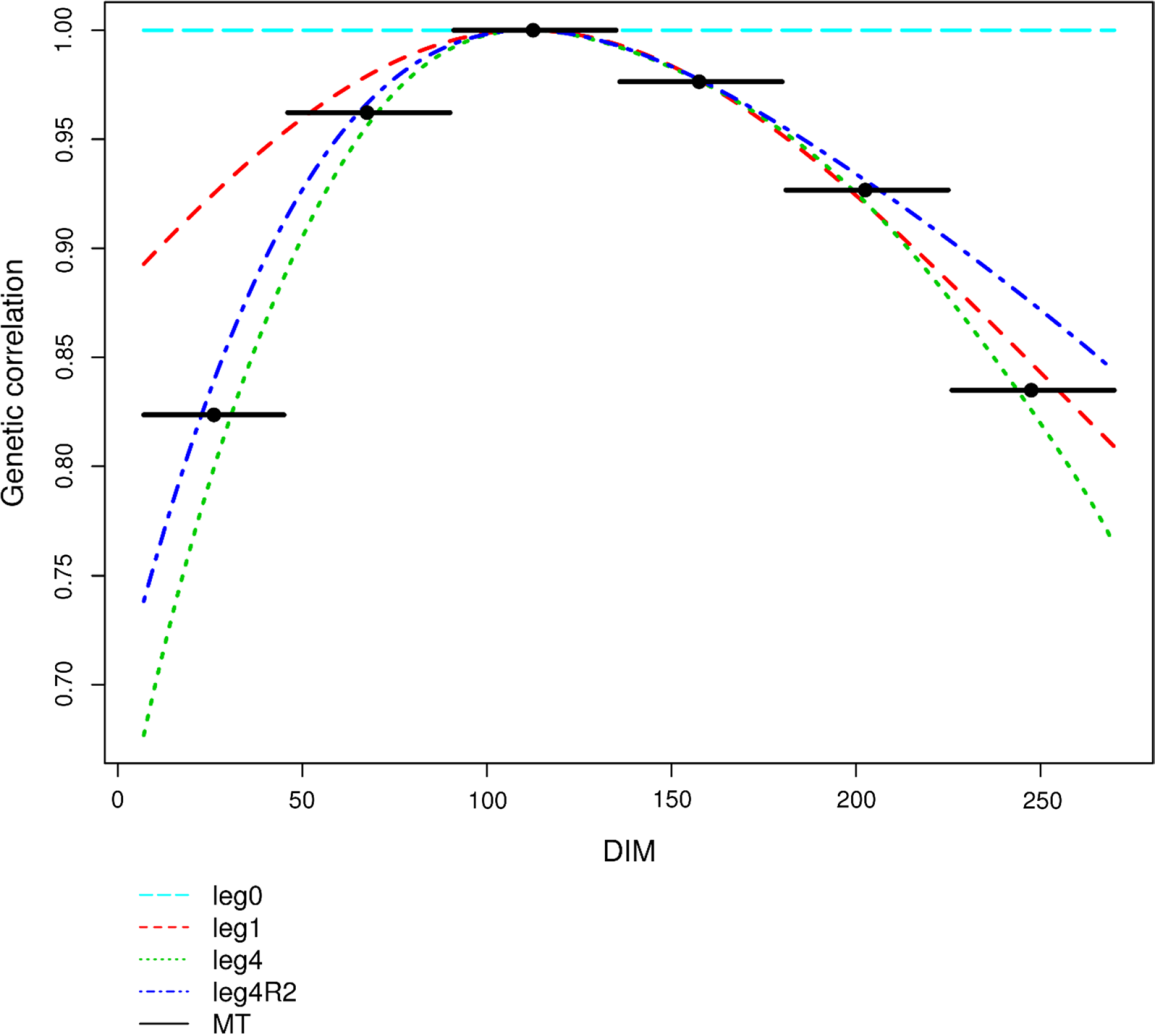
Genetic correlations of milk yields between DIM 111 and other DIM in Saanen goats

In order to compare the genetic correlations between periods obtained with the MT model and the genetic correlations between DIM obtained with the different RRM models, we selected the median day for each period of the MT model. Figure 4 and Additional file 8: Figure S5 show the genetic correlations of milk yield and protein content, respectively, at DIM 111 (i.e. the middle of the third period) with those at other DIM in the Saanen breed with the same models as those used for Fig. 2. The results are similar for both breeds. With model ${\text{leg}}0$, which assumes a constant genetic effect throughout lactation, the genetic correlation is equal to 1 between all DIM. This is also implicitly the case for the LACT model, which gives a global EBV for the whole lactation assuming a correlation of 1 between all DIM. The most similar genetic correlations are those estimated with the ${\text{leg}}4$ and MT models. As observed earlier with BIC, ${\text{leg}}4$ was the best model among all RRM. For all traits, the genetic correlations between any two periods estimated with the MT and RRM models were positive. The lowest correlation i.e. 0.44 was found with the MT model for milk yield in the Alpine breed between periods 1 and 6. The genetic correlations between DIM from the RRM* were fairly aligned to the pattern from the best full-rank model (${\text{leg}}4$) for all traits and both breeds.

Additional file 9: Figure S6 and Additional file 10: Figure S7 show the genetic correlations, estimated with the leg4 model, for all production traits between TD at DIM 40, which corresponds to the DIM at lactation peak, and at the other DIM, in both the Saanen and Alpine breeds. Genetic correlations for milk yield between the peak and the end of lactation were positive, i.e. ~ 0.5 in the Saanen and ~ 0.4 in the Alpine breed. In the Alpine breed, daily genetic correlations for fat and protein contents were higher (e.g. a correlation of ~ 0.7 for protein content between DIM 40 and at the end of lactation) than those for yield traits (e.g. a correlation of ~ 0.4 for milk yield between DIM 40 and at the end of lactation). For each trait, the genetic correlations between DIM were higher in the Saanen than the Alpine breed (e.g. correlations of ~ 0.4 (Alpine) and ~ 0.5 (Saanen) for milk yield between DIM 40 and end of lactation).

### Correlations between EBV for level of production and persistency

#### Within-trait

Table 3 presents the correlations between EBV for milk yield of bucks that were sires of the phenotyped goats (379 in Alpine and 324 in Saanen) in Saanen under the ${\text{leg}}1$, ${\text{leg}}4$ and ${\text{leg}}4{\text{R}}2$ models.

**Table 3:** Correlations between EBV for milk yield of buck sires of the recorded goats in Saanen

	LACT	${\text{SUM}}\_{\text{leg}}4$	${\text{PERS}}\_{\text{leg}}4$	${\text{a}}_{0} \_{\text{leg}}4$	${\text{a}}_{1} \_{\text{leg}}4$	${\text{SUM}}\_{\text{leg}}1$	${\text{a}}_{0} \_{\text{leg}}1$	${\text{a}}_{1} \_{\text{leg}}1$	${\text{SUM}}\_{\text{leg}}4{\text{R}}2$	${\text{b}}_{1} \_{\text{leg}}4{\text{R}}2$
${\text{SUM}}\_{\text{leg}}4$	0.99									
${\text{PERS}}\_{\text{leg}}4$	0.06	0.15								
${\text{a}}_{0} \_{\text{leg}}4$	0.99	1.00	0.15							
${\text{a}}_{1} \_{\text{leg}}4$	0.11	0.20	1.00	0.20						
${\text{SUM}}\_{\text{leg}}1$	0.99	1.00	0.15	1.00	0.20					
${\text{a}}_{0} \_{\text{leg}}1$	0.99	1.00	0.15	1.00	0.20	1.00				
${\text{a}}_{1} \_{\text{leg}}1$	0.10	0.19	1.00	0.19	0.99	0.19	0.19			
${\text{SUM}}\_{\text{leg}}4{\text{R}}2$	0.99	1.00	0.15	1.00	0.20	1.00	1.00	0.19		
${\text{b}}_{1} \_{\text{leg}}4{\text{R}}2$	0.99	1.00	0.18	1.00	0.23	1.00	1.00	0.22	1.00	
${\text{b}}_{2} \_{\text{leg}}4{\text{R}}2$	− 0.10	− 0.01	0.98	− 0.01	0.97	− 0.01	− 0.01	0.97	− 0.01	0.02

LACT, lactation model; SUM_${\text{leg}}x$, sum of the daily EBV of ${\text{leg}}x$; PERS_leg4, persistence calculated as in [18] from ${\text{leg}}4$; ${\text{a}}_{0} \_{\text{leg}}x$, regression coefficient for the 0th term of Legendre polynomial; ${\text{a}}_{1} \_{\text{leg}}x$, regression coefficient for the 1st term of the Legendre polynomial; ${\text{b}}_{1} \_{\text{leg}}4{\text{R}}2$, regression coefficient for the first eigenfunction; ${\text{b}}_{2} \_{\text{leg}}4{\text{R}}2$, regression coefficient for the second eigenfunction

We were able to evaluate the level of milk produced throughout lactation using the ${\text{LACT}}$ model, or by considering the ${\text{SUM}}\_{\text{leg}}x$ and ${\text{SUM}}\_{\text{leg}}4{\text{R}}2$ values, or the first coefficient of RRM (${\text{a}}_{0}$) or RRM* (${\text{b}}_{1}$). Correlations between these values reached a value of at least 0.99 for milk yield (Table 3) and all other traits, confirming that they characterize the same trait.

For the study of persistency, the ${\text{leg}}0$ and ${\text{LACT}}$ models were unsuitable because the resulting EBV were not DIM-dependent. Persistency could be evaluated by considering the ${\text{PERS}}\_{\text{leg}}4$ value, or the second coefficient of RRM (${\text{a}}_{1}$) and RRM* (${\text{b}}_{2}$). A correlation close to 1 was found between the ${\text{a}}_{1}$ coefficient of ${\text{leg}}4$ and ${\text{PERS}}\_{\text{leg}}4$ for milk yield in the Saanen breed. The correlation between ${\text{b}}_{2}$ and ${\text{PERS}}\_{\text{leg}}4$ was 0.98 for milk yield and 0.88 for protein yield.

The correlation between ${\text{SUM}}\_{\text{leg}}4$ and ${\text{PERS}}\_{\text{leg}}4$ was low for milk yield (i.e. 0.15, see Table 3) in Saanen and close to 0 in Alpine, but higher for protein content in both breeds (0.45 in Saanen and 0.58 in Alpine) (results not shown). The correlations between ${\text{a}}_{0}$ and ${\text{a}}_{1}$ from ${\text{leg}}1$ and ${\text{leg}}4$ were equal to 0.44, whereas those between ${\text{b}}_{1} \_{\text{leg}}4{\text{R}}2$ and ${\text{b}}_{2} \_{\text{leg}}4{\text{R}}2$ were equal to 0.01. The aim is to have a low genetic correlation between the level and the persistency because it allows selection of animals with a desired persistency throughout the lactation without changing the net-total lactation output.

#### Correlations between milk yield and other production traits

Table 4 reports the correlations between EBV of ${\text{SUM}}\_{\text{leg}}4$ (i.e. milk production level) and ${\text{PERS}}\_{\text{leg}}4$ (i.e. persistency) for milk yield and of ${\text{SUM}}\_{\text{leg}}4$ and ${\text{PERS}}\_{\text{leg}}4$ for the other traits for Saanen and Alpine bucks.

**Table 4:** Correlations between EBV for milk yield and the other traits for bucks

	Milk yield			
	Saanen		Alpine	
	${\text{SUM}}\_{\text{leg}}4$	${\text{PERS}}\_{\text{leg}}4$	${\text{SUM}}\_{\text{leg}}4$	${\text{PERS}}\_{\text{leg}}4$
Fat yield				
${\text{SUM}}\_{\text{leg}}4$	0.75	0.15	0.73	− 0.13
${\text{PERS}}\_{\text{leg}}4$	− 0.07	0.86	− 0.17	0.85
Protein yield				
${\text{SUM}}\_{\text{leg}}4$	0.90	0.18	0.88	− 0.04
${\text{PERS}}\_{\text{leg}}4$	0.16	0.94	0.02	0.93
Fat content				
${\text{SUM}}\_{\text{leg}}4$	− 0.20	0.06	− 0.33	− 0.14
${\text{PERS}}\_{\text{leg}}4$	− 0.36	− 0.10	− 0.32	− 0.17
Protein content				
${\text{SUM}}\_{\text{leg}}4$	− 0.33	0.01	− 0.44	− 0.10
${\text{PERS}}\_{\text{leg}}4$	− 0.29	− 0.45	− 0.28	− 0.58

For levels of production, the correlations between ${\text{SUM}}\_{\text{leg}}4$ for milk yield and ${\text{SUM}}\_{\text{leg}}4$ for the other traits averaged 0.75 for fat yield, 0.89 for protein yield, − 0.25 for fat content and around − 0.4 for protein content. The correlations between ${\text{SUM}}\_{\text{leg}}4$ for milk yield and persistencies of the other traits were moderate (all < 0.36 in absolute value). The correlations between milk yield persistency and ${\text{SUM}}\_{\text{leg}}4$ for the other traits were close to 0 (between − 0.14 and 0.18) for both breeds. The correlations between milk persistency and the other persistencies were very high (> 0.85) for protein and fat yields but very low for fat content (− 0.13, on average). However, the correlation with persistency of protein content was negative and relatively high (− 0.45 in Saanen and − 0.58 in Alpine). These quite high correlations indicate that a higher milk persistency is positively correlated with a higher-than-average protein content at the beginning of lactation. Because the correlation between fat content persistency and milk yield persistency was low (− 0.10 in Saanen and − 0.17 in Alpine) and because protein content at the beginning of lactation was higher than average, persistency is correlated with a reduced fat:protein ratio at the beginning of lactation.

As expected, the correlations of EBV between ${\text{b}}_{1} \_{\text{leg}}4{\text{R}}2$ and ${\text{b}}_{2} \_{\text{leg}}4{\text{R}}2$ for milk yield and ${\text{b}}_{1} \_{\text{leg}}4{\text{R}}2$ and ${\text{b}}_{2} \_{\text{leg}}4{\text{R}}2$ for the other traits (not shown) were very close to those obtained in Table 4. This result confirms that ${\text{b}}_{1} \_{\text{leg}}4{\text{R}}2$ is a measure of lactation production and ${\text{b}}_{2} \_{\text{leg}}4{\text{R}}2$ is a measure of persistency.

### Relative contribution of ${\text{b}}_{1}$ and ${\text{b}}_{2}$ coefficients to lactation production

**Fig. 5:**
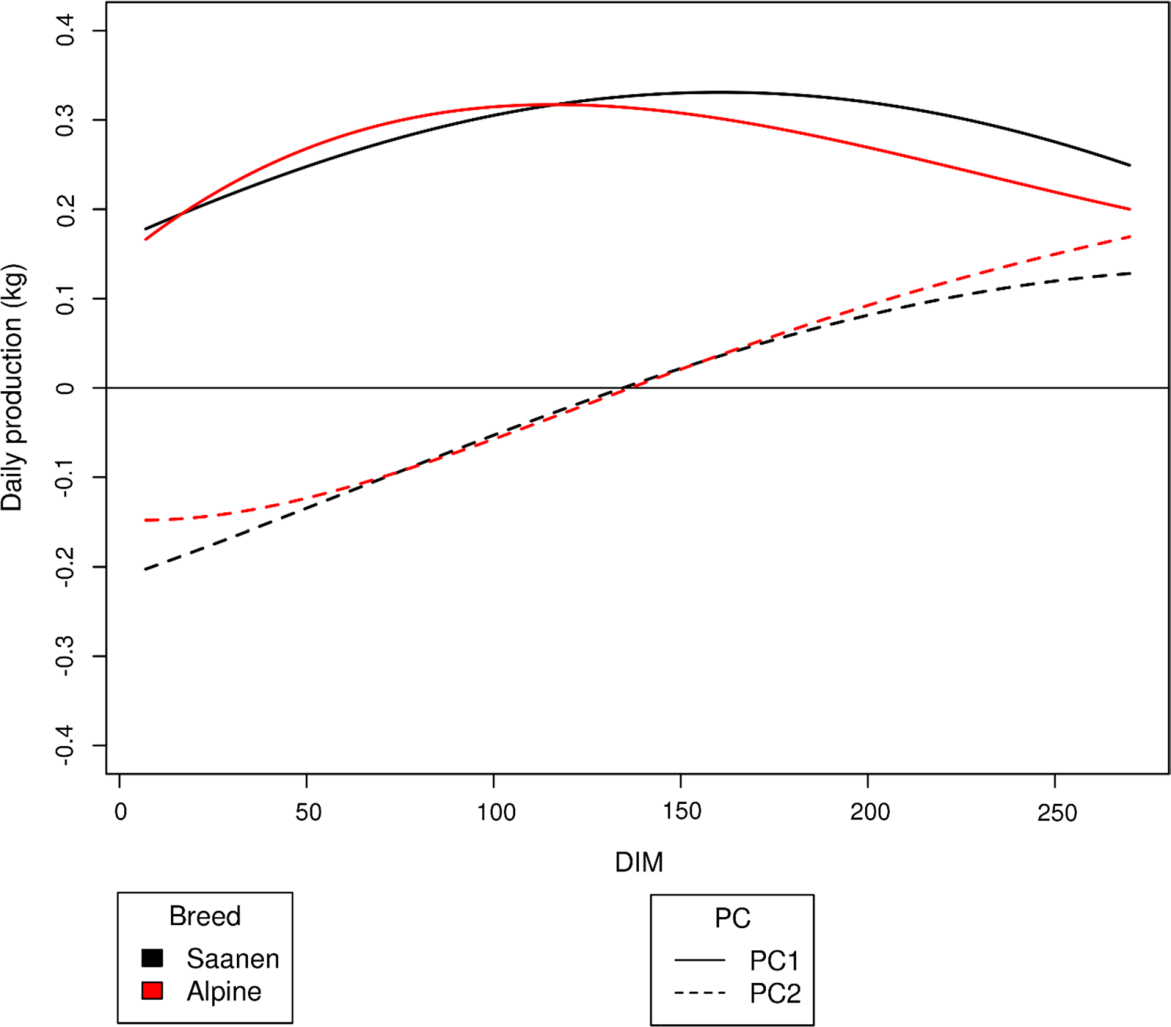
Contribution to daily milk yield of one genetic standard deviation for PC1 and PC2

Figure 5 and Additional file 11: Figure S8 show the relative contribution to lactation production of a change of one standard deviation in the ${\text{b}}_{1}$ (level) and ${\text{b}}_{2}$ (slope) coefficients for milk yield and protein content, respectively, with ${\text{leg}}4{\text{R}}2$. An increase of one genetic standard deviation for ${\text{b}}_{1}$ leads to an increased production level throughout lactation, with a higher increase at the end of lactation for Saanen compared to Alpine goats. This study on the one-standard-deviation increase of ${\text{b}}_{1}$ and ${\text{b}}_{2}$ allows to analyze the potential result of a selection on these two traits. As a consequence of the one-standard-deviation increase of ${\text{b}}_{1}$, the sum of the added daily milk productions represents 76 kg of milk for Saanen and 71.1 kg for Alpine goats, which is relatively consistent with the ${\text{LACT}}$ model (genetic standard deviation equal to 92 kg in Saanen and 85 kg in Alpine goats). At the beginning of lactation, increasing the persistency (${\text{b}}_{2}$) by one standard deviation reduces production by 12.9 kg until DIM 129 and then increases it by 10.5 kg at the end of the lactation for Saanen. For protein content, a gain of one standard deviation of ${\text{b}}_{1}$ increases average production more in Alpine than in Saanen goats. A loss of one standard deviation in protein content persistency leads to an increase of 1 g/kg at the beginning of lactation and a loss of 1 g/kg at the end of lactation.

**Fig. 6:**
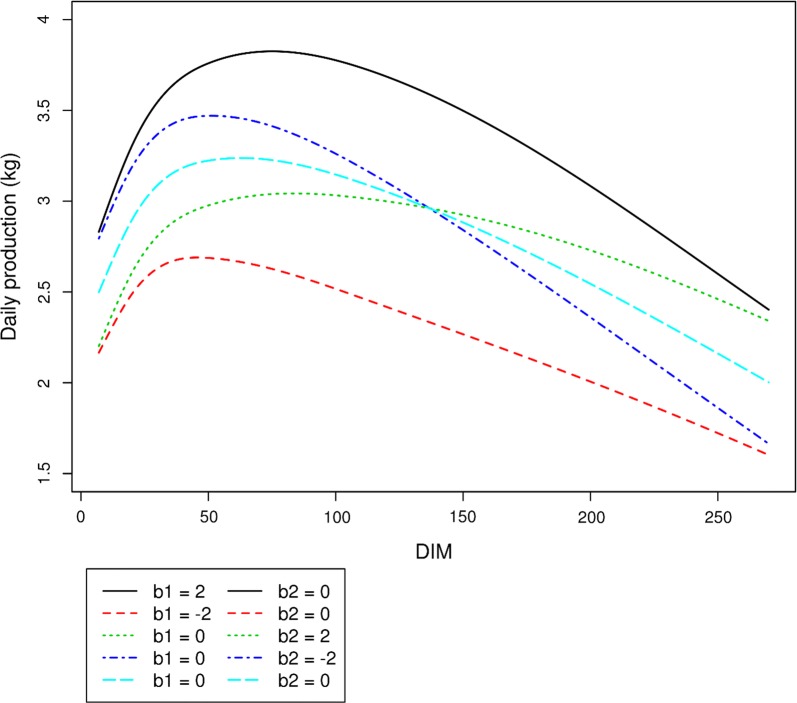
Genetic value of extreme Alpine goats for milk yield added to the mean production curve (${\text{b}}_{1}$ expressed in standard deviation; ${\text{b}}_{2}$ expressed in standard deviation)

As an illustration, Fig. 6 displays the genetic daily EBV added to the average standard production curve, for two extreme-value goats for global production level and two extreme-value goats for milk yield persistency. Additional file 12: Figure S9 shows the same results but for protein yield.

## Discussion

We estimated various genetic parameters in a large population of goats that had been regularly measured for five major dairy traits throughout lactation since 1995. This estimation was done by using a random regression model for the first time in France for the two main goat breeds Alpine and Saanen, which represent 97% of the goats recorded in France. Connectedness between the large number of herds was ensured by using phenotypes from daughters of sires from artificial insemination (379 and 324 in the Alpine and Saanen breed, respectively).

The fixed effects, i.e. age at kidding, month of kidding, dry-period length and gestation stage, were modeled with splines in the RRM model. In a previous study [11], we showed that these factors had an impact on the shape of the lactation curve, and that there was no interaction between these effects and year of lactation. These results were consistent with other studies in dairy cows [14, 23]. Unlike other studies in dairy goats [4, 5], we did not use an effect on litter size since this information was not available in our dataset, but Mucha et al. [1] found no impact of this effect on EBV.

We applied a principal component analysis of the variance–covariance matrix of the most complex model, which showed that it was possible to associate biological significance with each PC. As in our study, van der Werf et al. [17], Olori et al. [24], Druet et al. [14] and Togashi and Lin [25] highlighted that the first eigenfunction was linked with the average production level throughout lactation, the second eigenfunction was associated with persistency, and the third eigenfunction opposed production around the middle of lactation against production at the beginning and end of lactation. The high percentages of variances explained by the first three PC (and even the two first PC), and the desire to reduce the overall dimension of the model in routine evaluations, pointed at the need to test a rank reduction of the variance–covariance matrix.

Implementing a RRM for genetic evaluation requires cumbersome testing of the best tradeoffs between model fit and complexity. Pool et al. [26] compared the residual variances of different RRM on cow data and found, as we did here, that the most suitable model for TD milk production traits was ${\text{leg}}4$. The use of other criteria (BIC and Pearson correlation coefficients) also confirmed that ${\text{leg}}4$ was the most suitable model for TD milk production traits. The ${\text{leg}}x{\text{R}}3$ reductions (for $x = \left\{ {3.4} \right\}$) had a better fit to the data than ${\text{leg}}x{\text{R}}2$ although ${\text{leg}}x{\text{R}}2$ reductions led to a better adjustment to the data compared to other more concise models such as ${\text{leg}}0$ and ${\text{leg}}1$, especially for milk yield. This rank reduction facilitates the extension of the model to subsequent lactations 2 and 3 in order to construct a genetic evaluation. Indeed, this model extension has to take into account how genetic correlations between first and following parities will probably differ from 1 (around 0.7 for milk yield) as found by several studies in dairy goats [1, 4].

For all traits, heritability estimates from the ${\text{LACT}}$ model and for the regression coefficients from RRM and RRM* were close to those reported by Rupp et al. [27] for the same five traits in French Alpine and Saanen goats in first lactation. The evolution of the heritability throughout lactation for each trait was similar between RRM and RRM*. In Norwegian goats, Andonov et al. [6] showed that the heritability of milk yield estimated with the MT model increased up to a maximum at mid-lactation (0.26) and then decreased, which agrees with our observations. The evolution of the heritability throughout the DIM period found here for milk yield differed from that reported in other studies using RRM in goats. Menéndez-Buxadera et al. [4] found a maximum heritability of 0.24 at the beginning of lactation, then a decrease with DIM in Murciano-Granadina goats whereas Zumbach et al. [5] found a maximum heritability of 0.4 and then a decrease with DIM in six German breeds. Andonov et al. [6] found a maximum heritability of 0.33 at ~ DIM 155, in a Norwegian goat breed and Mucha et al. [1] found a maximum heritability of 0.45 at ~ DIM 220, in a crossbred population including three goat breeds: Alpine, Saanen, and Toggenburg. The mean heritability estimates for protein yield found here (~ 0.2) was in agreement with that reported by Muños-Mejias et al. [3] for goats of the Florida breed. This was not the case for fat yield, for which we found a higher heritability (~ 0.2) than that for the Florida breed (~ 0.15). Moreover, Muños-Mejias et al. [12] showed that the estimated heritabilities for fat and protein yield for Florida goats were higher at the end of lactation, which was not the case in our study. For protein and fat contents, the estimated heritabilities reported in Muños-Mejías et al. [3] and Andonov et al. [6] were lower than those found here, and followed a different shape, i.e. they increased with DIM. The RRM* made it possible to calculate the heritability of persistency for each trait [21]. The heritabilities of persistency for milk yield calculated from RRM* were close to those reported by Cole and VanRaden [8] in cattle, who also calculated milk yield persistency ensuring that it was not correlated with milk yield level. Menéndez-Buxadera et al. [4] reported a heritability of milk yield persistency 0.208, but the correlation with lactation yield level is unknown.

As in our study, Mucha et al. [1] found that all the genetic correlations for milk yield between two periods in the trajectory fitted in the RRM models were always positive. This indicates that selection of animals based on any daily EBV will yield positive responses for all the other days in the lactation curve. Also in agreement with our results, Muñoz-Mejías et al. [3], Andonov et al. [6] and Menéndez-Buxadera et al. [4] found that genetic correlations between days were higher for fat and protein contents than for yield traits. The moderate genetic correlations for milk yield between the peak and end of lactation indicated genetic variability in the level of milk production between the peak and end of lactation, and therefore a genetic variability for milk persistency. For all traits, the between-day genetic correlations were similar with RRM or RRM*.

We showed that correlations between EBV from RRM and RRM* were close to 1 for both average and persistency of yield as reported in Leclerc et al. [28] who compared EBV from a complete and a reduced model. The advantages of the reduced model are the smaller size of the variance–covariance matrices and the zero correlation between EBV for lactation yield (${\text{b}}_{1}$) and persistency (${\text{b}}_{2}$) by construction. This allows selection of animals with a desired persistency throughout the lactation without changing the net-total lactation output. Model ${\text{leg}}1$ does not have this advantage as the correlation between mean level and persistency is high in that model. The large difference in lactation trajectory between extreme animals suggests that it can be valuable to consider persistency in selection. The form of the first eigenfunction (Fig. 5) is interesting because it represents the pattern of how a goat produces milk throughout lactation from its genetic makeup. The eigenfunction coordinates were higher at the end of the lactation period for the Saanen breed than for Alpine breed, confirming the observations of Arnal et al. [11] who showed a better persistency for Saanen goats than Alpine goats. This is also evidenced by the higher correlation between SUM_leg4 and ${\text{PERS}}\_{\text{leg}}4$ for the Saanen compared to Alpine goats (0.15 in Saanen vs. 0 in Alpine) as well as the higher genetic correlations between DIM in Saanen compared to Alpine goats.

The correlations of the production level for milk yield with the production level for the other traits were very close to those reported by Bélichon et al. [29] on total lactation traits (0.90 for protein yield and 0.76 for fat yield). For protein content and fat content, Bélichon et al. [29] found slightly lower correlations, i.e. − 0.28 for protein content and − 0.13 for fat content while we found 0.39 and − 0.27, respectively, in our study. The correlations between milk yield persistency and the production level for the other traits were weak or close to 0 for both breeds, indicating that milk yield persistency can be selected for with no impact on content-related traits. Furthermore, the difference in the correlations observed here between milk persistency and protein content persistency on the one hand, and between milk persistency and fat content persistency on the other hand, indicates that a goat with a high milk persistency will tend to have a lower fat:protein ratio in early lactation. Several studies in dairy cattle [13, 30, 31] showed that a high fat:protein ratio was associated with a negative energy balance, subclinical mastitis, and poor fertility. These results point out to the potential value of lactation persistency in breeding schemes.

Finally, the development of a test-day model for milk production opens up new perspectives. For example, the study of the genetic relationship between persistency and other traits such as longevity or fertility could help to explain the negative correlation between high production and fitness. The estimated fixed effects of such test-day models, especially the herd-test-day effect, also offer producers important clues on the impact of herd management on these traits. For example, herd test-day estimates can be compared between farms under similar systems in terms of mean and variability throughout the year [23].

## Conclusions

In this paper, we show that the genetic parameters obtained with a test-day model using a fourth-order Legendre polynomial (${\text{leg}}4$) for the genetic and permanent environmental components and a multi-trait model are consistent. However, this kind of model is complex and computationally demanding. Given that the aim was to develop TD genetic evaluations for traits in selection schemes (milk production traits and somatic cell score) over several parities and possibly including genomic information, a simpler model is necessary. We found that reducing the genetic and permanent environment (co)variance matrices of ${\text{leg}}4$ to its first two PC (${\text{leg}}4{\text{R}}2$) was a satisfactory compromise, which accurately approximates genetic parameters and EBV under the complete model. This reduced model can give EBV for total lactation milk yield and persistency that are nearly independent (correlation close to 0). This negligible correlation is appealing because it allows to select animals with a desired lactation shape independently from selection for total lactation production. Therefore, for the extension of the TD models to several lactations, we consider that ${\text{leg}}4{\text{R}}2$ is more appropriate for implementation than the complete model ${\text{leg}}4$.

## Additional files

**Additional file 1: Table S1.** Proportion (%) of genetic variance explained by the first three principal components (PC) using leg4.

**Additional file 2: Figure S1.** Coordinates of the first three eigenfunctions of the test-day genetic covariance matrix computed with leg4 in Saanen goats.

**Additional file 3: Table S2.** Pearson correlation coefficients (ρ) between observed and predicted values with the complete and reduced models. MT: multitrait model; leg*x*: Legendre polynomial of order *x*; leg*x*Rz: Legendre polynomial of order* x* reduced to order* z*.

**Additional file 4: Figure S2.** Evolution of residual variances with DIM for protein content in Alpine goats (full-rank models: leg0, leg1, leg2, leg4; reduced model: leg4R2).

**Additional file 5: Table S3.** Whole-lactation heritabilities from the LACT model and heritabilities (h_wl) calculated as in [22] in Saanen and Alpine goats.

**Additional file 6: Figure S3.** Daily estimated heritabilities for protein content in Saanen goats (full-rank models: leg0, leg1, leg4; best reduced model: leg4R2 and the MT model).

**Additional file 7: Figure S4.** Evolution of heritabilities with DIM in Alpine goats with the leg4 model.

**Additional file 8: Figure S5.** Genetic correlations of protein contents between DIM 111 and other DIM in Saanen goats.

**Additional file 9: Figure S6.** Genetic correlations between DIM 40 and other DIM in Alpine goats from the leg4 model.

**Additional file 10: Figure S7.** Genetic correlations between DIM 40 and other DIM in Saanen goats from the leg4 model.

**Additional file 11: Figure S8.** Contribution to daily protein content of one genetic standard deviation for PC1 and PC2.

**Additional file 12: Figure S9.** EBV of Alpine goats added to the mean production of the population for protein content (b_1_ expressed in standard deviation; b_2_ expressed in standard deviation).

## Data Availability

The datasets analyzed in this study are not publicly available since they were partially produced by private professional partnerships.
