# Supplementary material for: Genetic parameters for first lactation dairy traits in the Alpine and Saanen goat breeds using a random regression test-day model
Source: Genet Sel Evol. 2019 Aug 13;51:43. doi: 10.1186/s12711-019-0485-3 (PMC6693143; doi:10.1186/s12711-019-0485-3)
Supplement: Supplementary file 1 — Additional file 1: Table S1. Proportion (%) of genetic variance explained by the first three principal components (PC) using leg4. [file 12711_2019_485_MOESM1_ESM.docx]

|  | Milk yield | | Fat yield | | Protein yield | | Fat content | | Protein content | |
| --- | --- | --- | --- | --- | --- | --- | --- | --- | --- | --- |
|  | Saanen | Alpine | Saanen | Alpine | Saanen | Alpine | Saanen | Alpine | Saanen | Alpine |
| PC1 | 87.5 | 84.4 | 87.6 | 83.1 | 89.2 | 85.4 | 92.7 | 90.7 | 90.3 | 91.7 |
| PC2 | 10.6 | 12.1 | 11 | 12.3 | 9.1 | 9.9 | 4.8 | 6.6 | 7.5 | 6.9 |
| PC3 | 1.5 | 2.9 | 1.1 | 4.2 | 1.2 | 4 | 1.8 | 2.2 | 1.8 | 1 |
|  |  |  |  |  |  |  |  |  |  |  |
| sum of first 2 PC | 98.1 | 96.5 | 98.6 | 95.4 | 98.3 | 95.3 | 97.5 | 97.3 | 97.8 | 98.6 |
| sum of first 3 PC | 99.6 | 99.4 | 99.7 | 99.6 | 99.5 | 99.3 | 99.3 | 99.5 | 99.6 | 99.6 |
