# Supplementary figures and images for: Genetic parameters for first lactation dairy traits in the Alpine and Saanen goat breeds using a random regression test-day model
Source: Genet Sel Evol. 2019 Aug 13;51:43. doi: 10.1186/s12711-019-0485-3 (PMC6693143; doi:10.1186/s12711-019-0485-3)

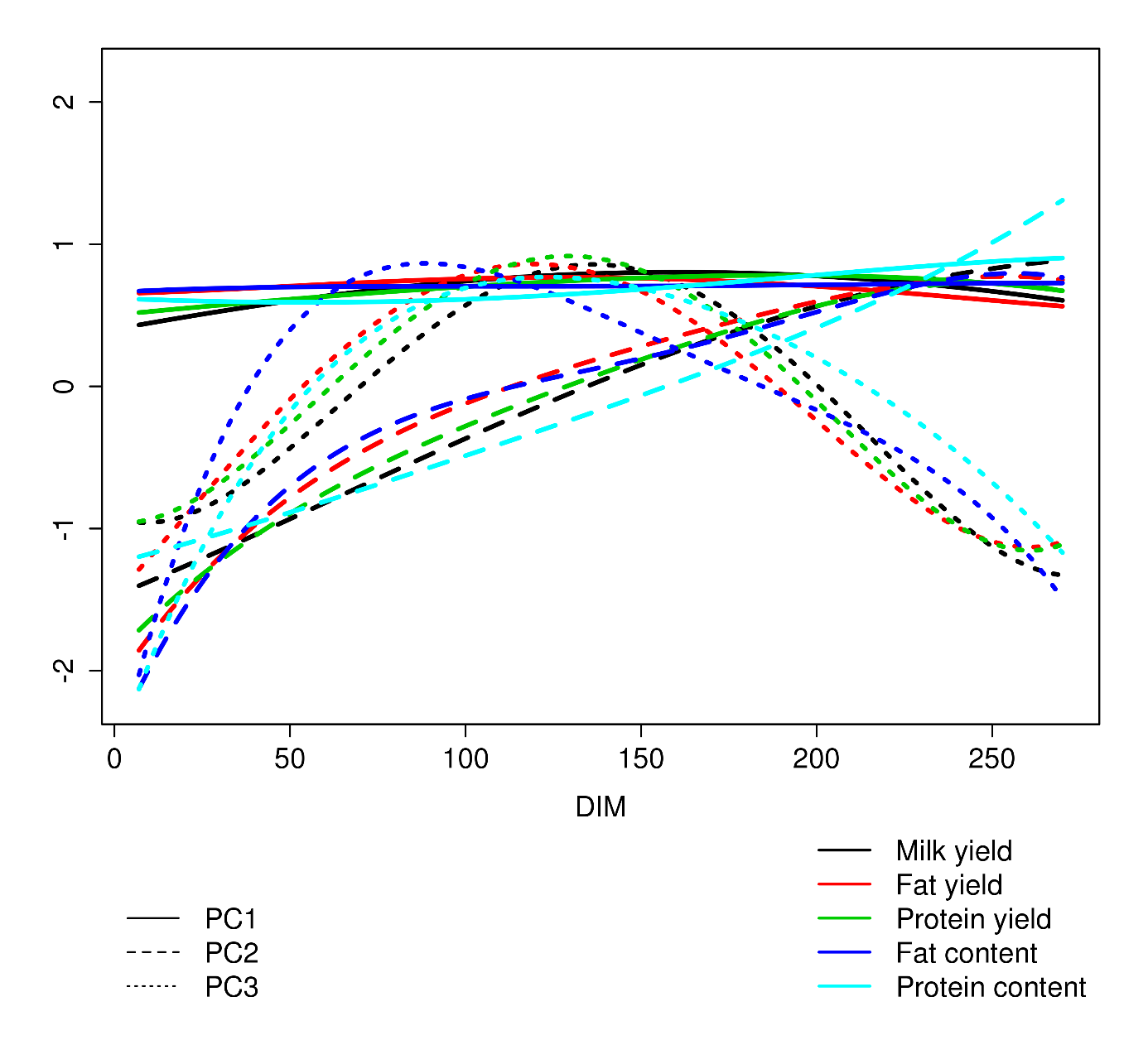

Supplement: Supplementary file 2 — Additional file 2: Figure S1. Coordinates of the first three eigenfunctions of the test-day genetic covariance matrix computed with leg4 in Saanen goats. [file 12711_2019_485_MOESM2_ESM.docx]

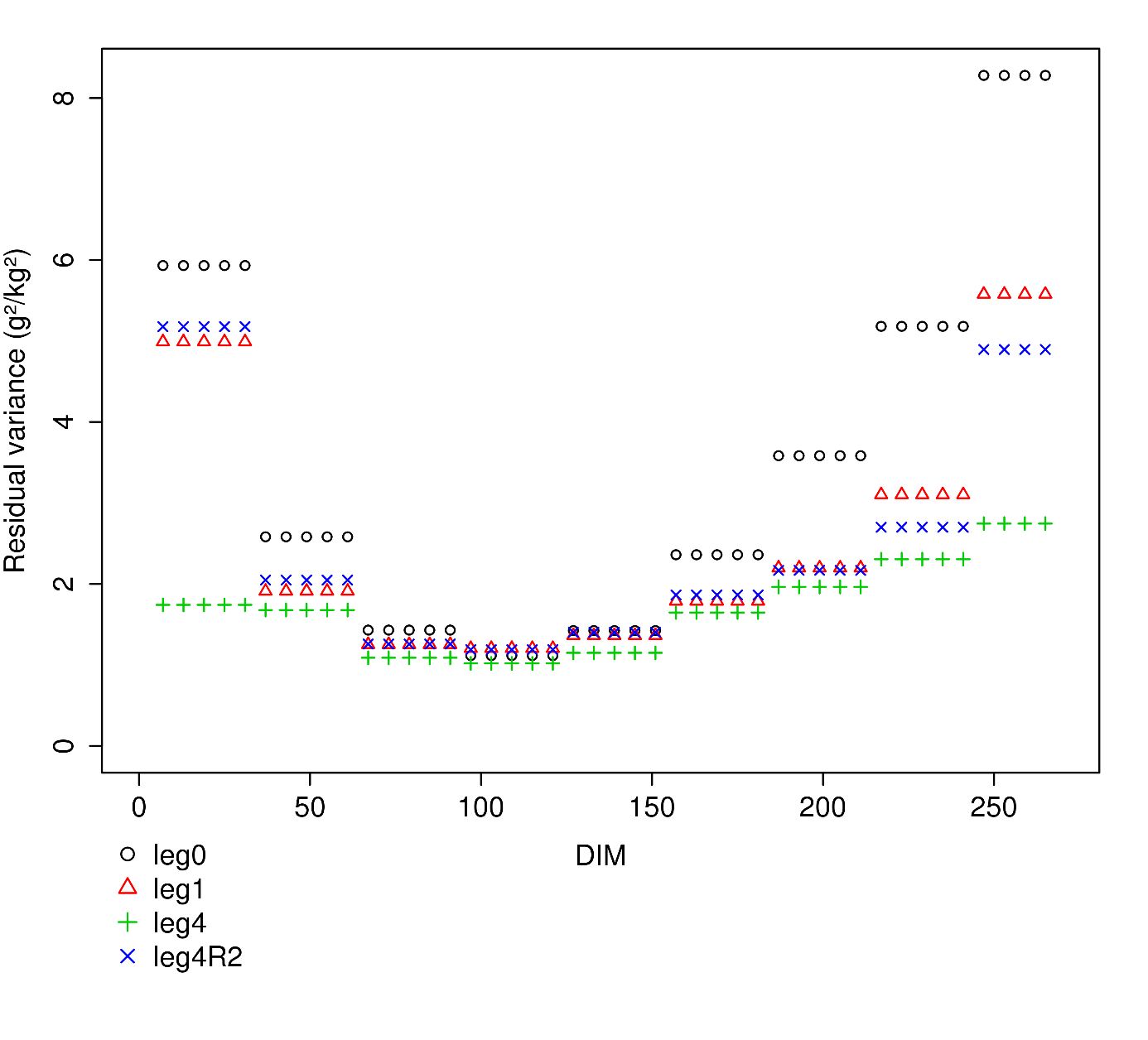

Supplement: Supplementary file 4 — Additional file 4: Figure S2. Evolution of residual variances with DIM for protein content in Alpine goats (full-rank models: leg0, leg1, leg2, leg4; reduced model: leg4R2). [file 12711_2019_485_MOESM4_ESM.docx]

*
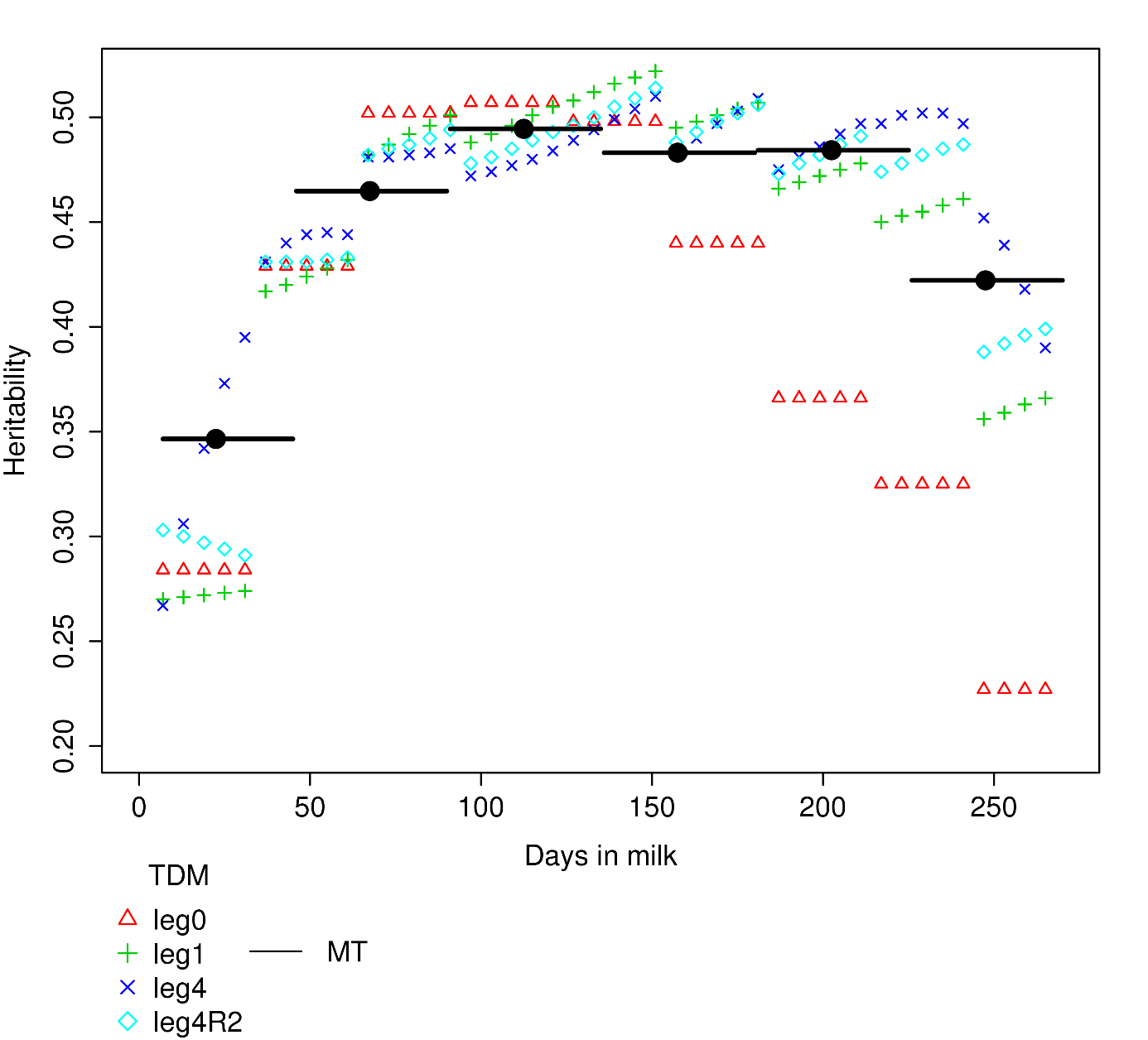
*

Supplement: Supplementary file 6 — Additional file 6: Figure S3. Daily estimated heritabilities for protein content in Saanen goats (full-rank models: leg0, leg1, leg4; best reduced model: leg4R2 and the MT model). [file 12711_2019_485_MOESM6_ESM.docx]

*
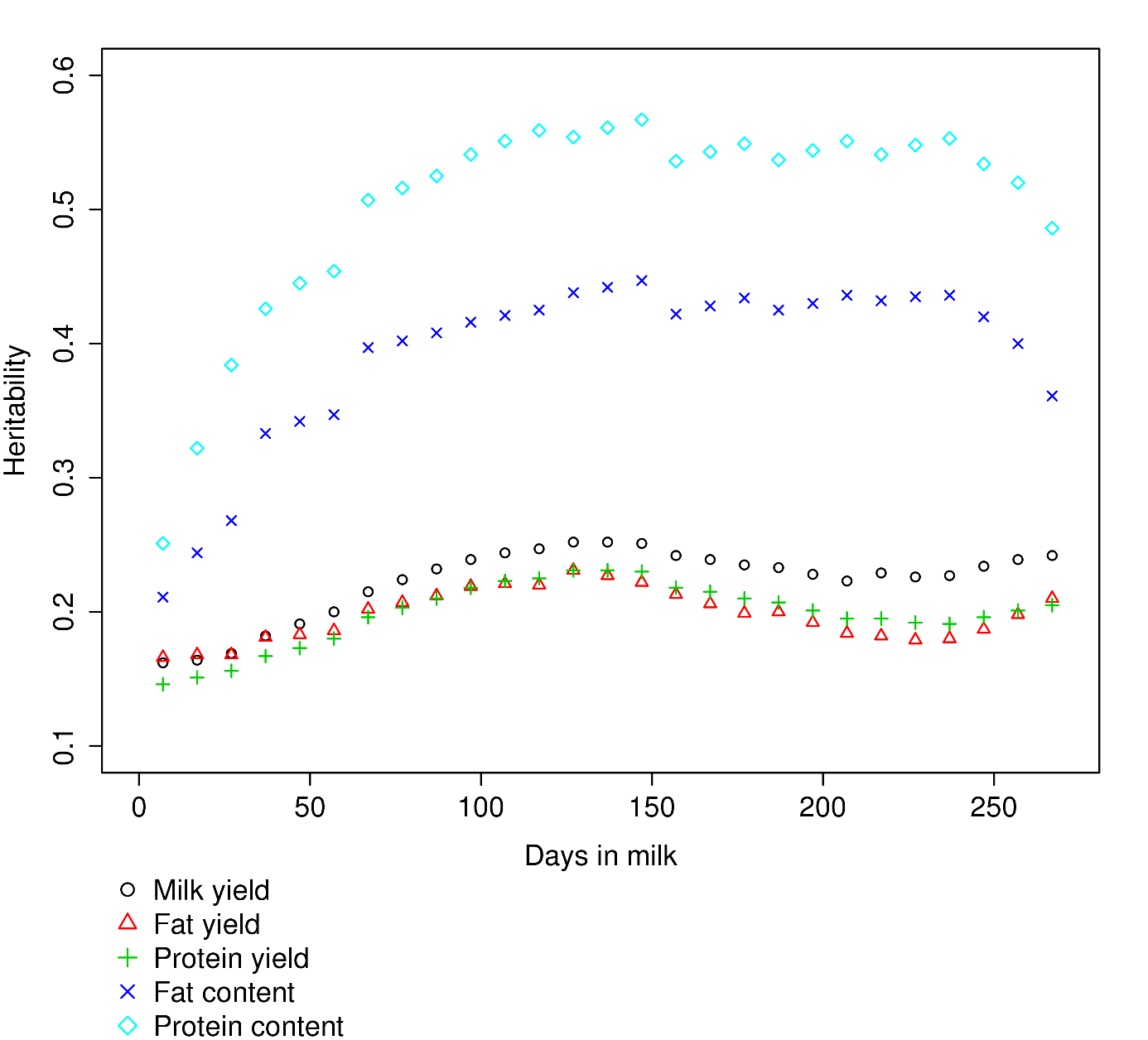
*

Supplement: Supplementary file 7 — Additional file 7: Figure S4. Evolution of heritabilities with DIM in Alpine goats with the leg4 model. [file 12711_2019_485_MOESM7_ESM.docx]

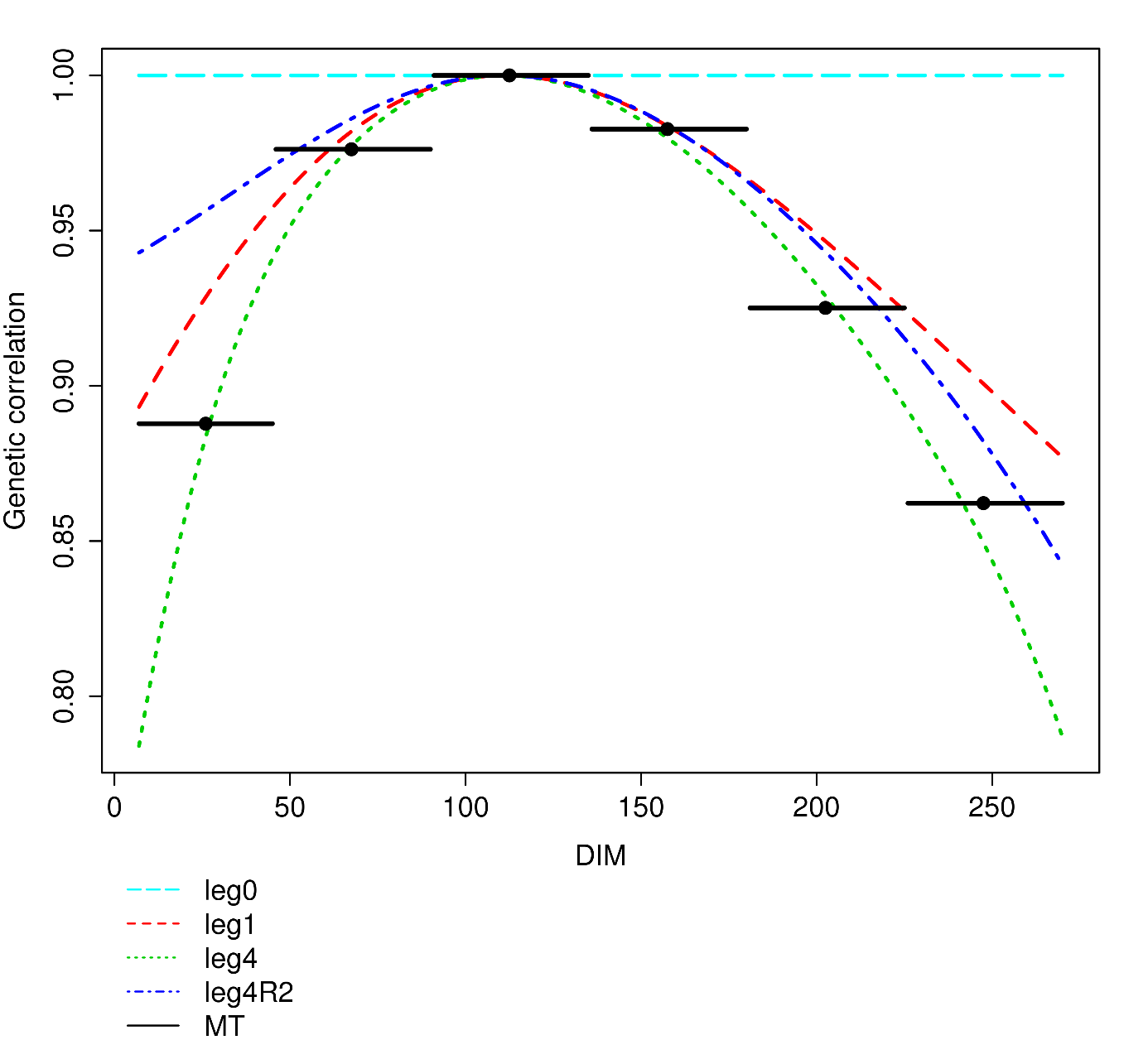

Supplement: Supplementary file 8 — Additional file 8: Figure S5. Genetic correlations of protein contents between DIM 111 and other DIM in Saanen goats. [file 12711_2019_485_MOESM8_ESM.docx]

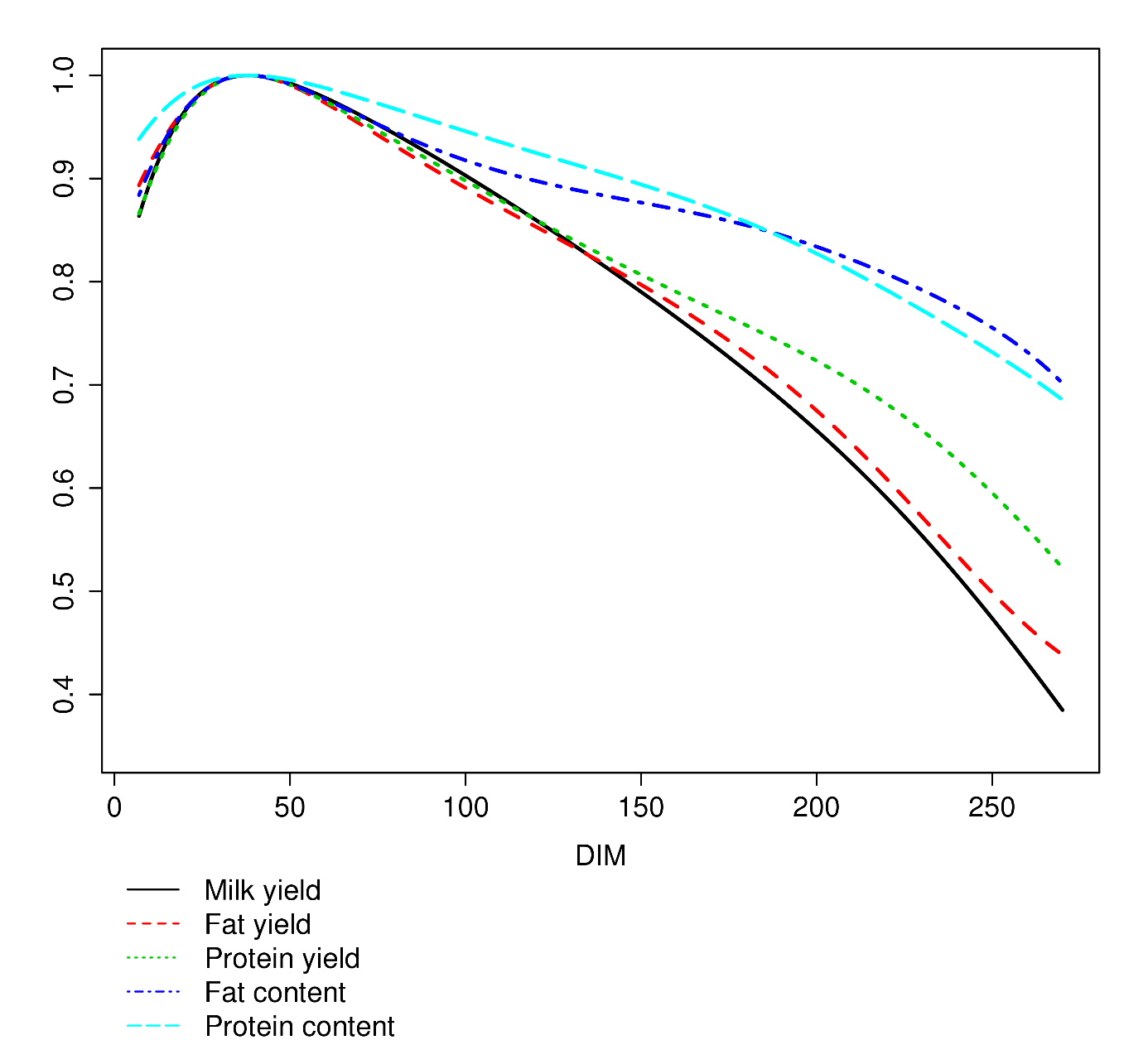

Supplement: Supplementary file 9 — Additional file 9: Figure S6. Genetic correlations between DIM 40 and other DIM in Alpine goats from the leg4 model. [file 12711_2019_485_MOESM9_ESM.docx]

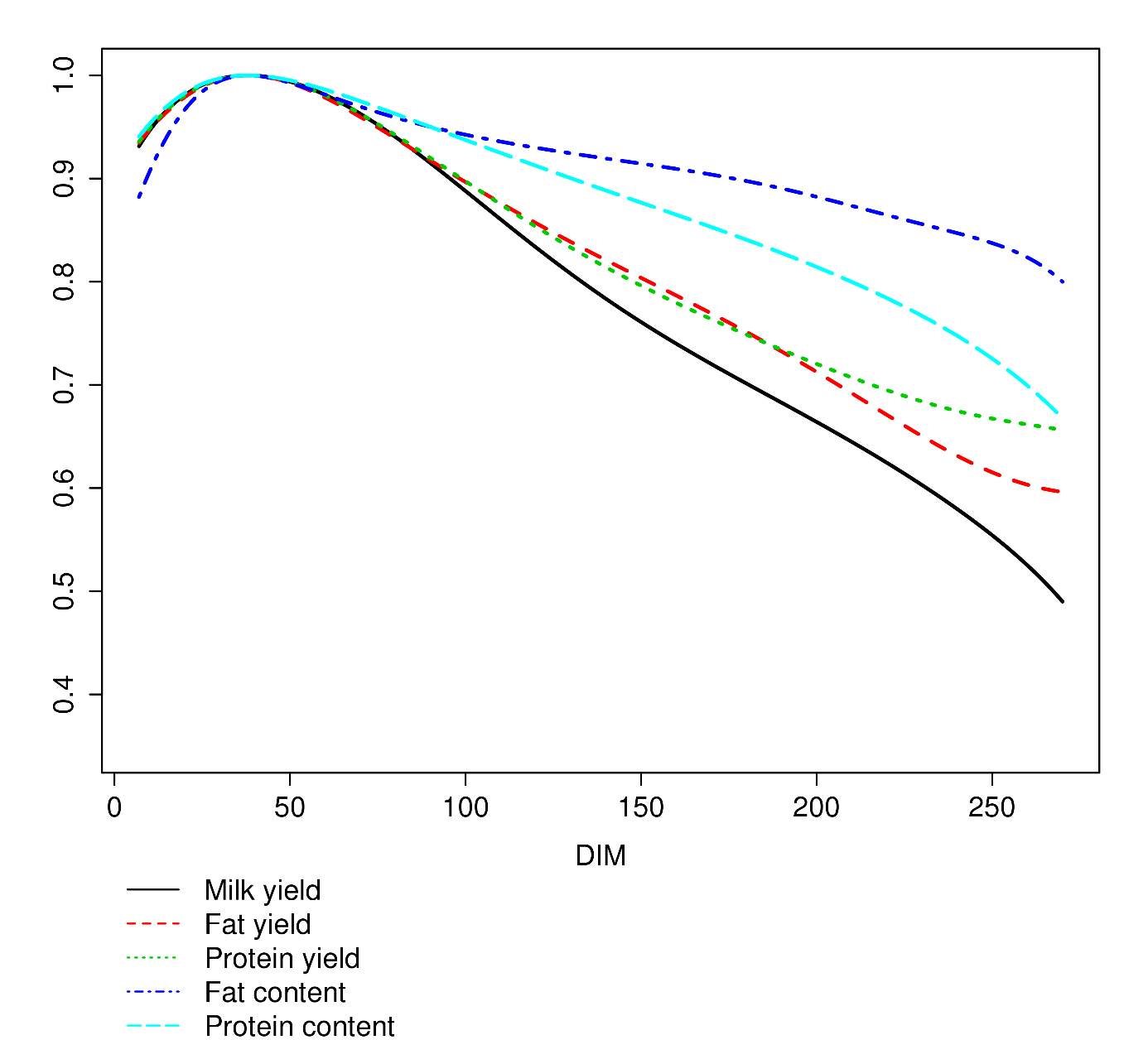

Supplement: Supplementary file 10 — Additional file 10: Figure S7. Genetic correlations between DIM 40 and other DIM in Saanen goats from the leg4 model. [file 12711_2019_485_MOESM10_ESM.docx]

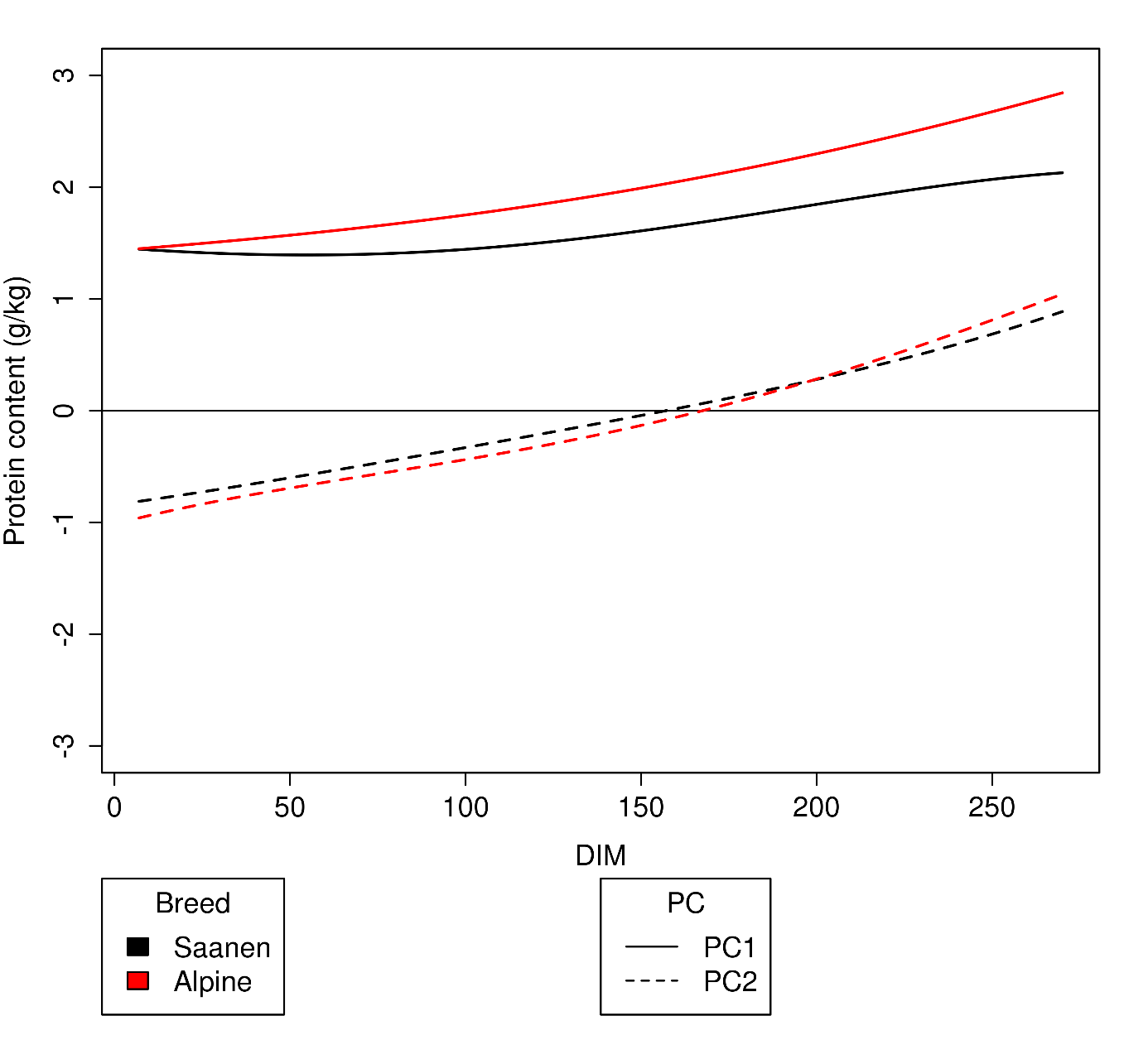

Supplement: Supplementary file 11 — Additional file 11: Figure S8. Contribution to daily protein content of one genetic standard deviation for PC1 and PC2. [file 12711_2019_485_MOESM11_ESM.docx]

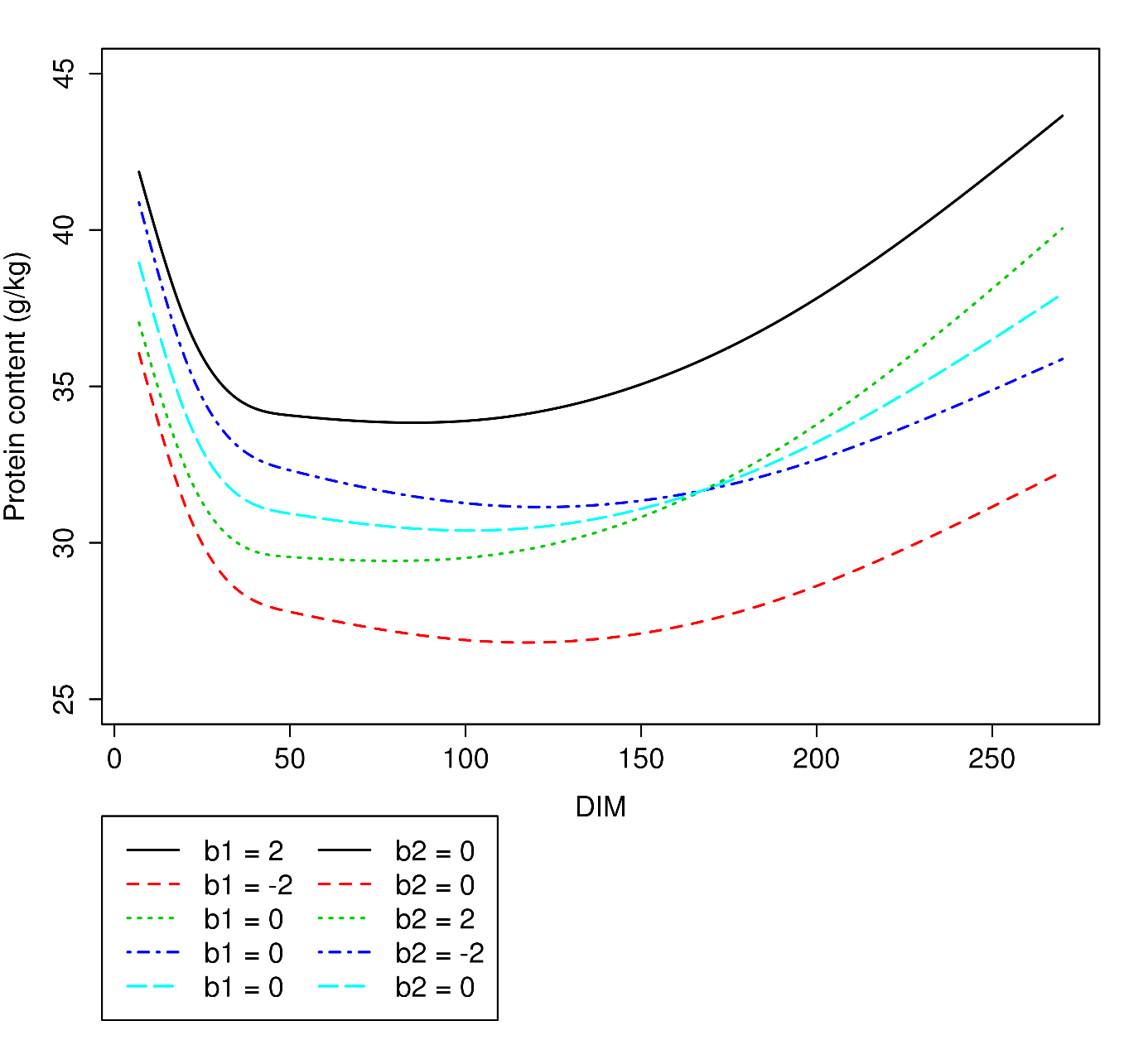

Supplement: Supplementary file 12 — Additional file 12: Figure S9. EBV of Alpine goats added to the mean production of the population for protein content (b1 expressed in standard deviation; b2 expressed in standard deviation). [file 12711_2019_485_MOESM12_ESM.docx]
