# Supplementary material for: Genetic parameters for first lactation dairy traits in the Alpine and Saanen goat breeds using a random regression test-day model
Source: Genet Sel Evol. 2019 Aug 13;51:43. doi: 10.1186/s12711-019-0485-3 (PMC6693143; doi:10.1186/s12711-019-0485-3)
Supplement: Supplementary file 3 — Additional file 3: Table S2. Pearson correlation coefficients (ρ) between observed and predicted values with the complete and reduced models. MT: multitrait model; legx: Legendre polynomial of order x; legxRz: Legendre polynomial of order x reduced to order z. [file 12711_2019_485_MOESM3_ESM.docx]

|  |  | **Saanen** | | | | |  | **Alpine** | | | | |
| --- | --- | --- | --- | --- | --- | --- | --- | --- | --- | --- | --- | --- |
|  |  | **Milk yield** | **Fat yield** | **Protein yield** | **Fat content** | **Protein content** |  | **Milk yield** | **Fat yield** | **Protein yield** | **Fat content** | **Protein content** |
|  | **MT** | 0.81 | 0.83 | 0.82 | 0.85 | 0.88 |  | 0.81 | 0.81 | 0.80 | 0.85 | 0.90 |
| **Complete model** | **leg0** | 0.91 | 0.89 | 0.90 | 0.87 | 0.90 |  | 0.91 | 0.88 | 0.90 | 0.87 | 0.91 |
|  | **leg1** | 0.94 | 0.92 | 0.93 | 0.90 | 0.93 |  | 0.94 | 0.92 | 0.93 | 0.89 | 0.94 |
|  | **leg2** | 0.96 | 0.94 | 0.95 | 0.91 | 0.95 |  | 0.95 | 0.93 | 0.94 | 0.90 | 0.96 |
|  | **leg3** | 0.96 | 0.94 | 0.96 | 0.92 | 0.96 |  | 0.96 | 0.94 | 0.95 | 0.92 | 0.97 |
|  | **leg4** | 0.96 | 0.95 | 0.96 | 0.93 | 0.97 |  | 0.96 | 0.95 | 0.96 | 0.93 | 0.97 |
| **Reduced model** | **leg2r2** | 0.94 | 0.93 | 0.94 | 0.90 | 0.93 |  | 0.94 | 0.92 | 0.93 | 0.89 | 0.94 |
|  | **leg3r2** | 0.94 | 0.93 | 0.94 | 0.90 | 0.93 |  | 0.94 | 0.92 | 0.93 | 0.89 | 0.95 |
|  | **leg4r2** | 0.94 | 0.93 | 0.94 | 0.90 | 0.93 |  | 0.94 | 0.92 | 0.93 | 0.89 | 0.95 |
|  | **leg3r3** | 0.96 | 0.94 | 0.95 | 0.92 | 0.95 |  | 0.96 | 0.94 | 0.94 | 0.91 | 0.96 |
|  | **leg4r3** | 0.96 | 0.94 | 0.95 | 0.92 | 0.95 |  | 0.96 | 0.94 | 0.94 | 0.91 | 0.96 |
