# Supplementary material for: Genetic parameters for first lactation dairy traits in the Alpine and Saanen goat breeds using a random regression test-day model
Source: Genet Sel Evol. 2019 Aug 13;51:43. doi: 10.1186/s12711-019-0485-3 (PMC6693143; doi:10.1186/s12711-019-0485-3)
Supplement: Supplementary file 5 — Additional file 5: Table S3. Whole-lactation heritabilities from the LACT model and heritabilities (h_wl) calculated as in [22] in Saanen and Alpine goats. [file 12711_2019_485_MOESM5_ESM.docx]

|  |  | **Saanen** | | | | |  | **Alpine** | | | | |
| --- | --- | --- | --- | --- | --- | --- | --- | --- | --- | --- | --- | --- |
|  |  | **Milk yield** | **Fat yield** | **Protein yield** | **Fat content** | **Protein content** |  | **Milk yield** | **Fat yield** | **Protein yield** | **Fat content** | **Protein content** |
|  | **Lact** | 0.29 | 0.33 | 0.30 | 0.6 | 0.62 |  | 0.26 | 0.26 | 0.25 | 0.65 | 0.66 |
|  |  |  |  |  |  |  |  |  |  |  |  |  |
| **Complete model** | **Leg0** | 0.32 | 0.39 | 0.35 | 0.69 | 0.64 |  | 0.3 | 0.32 | 0.3 | 0.77 | 0.7 |
|  | **Leg1** | 0.32 | 0.38 | 0.35 | 0.68 | 0.65 |  | 0.29 | 0.31 | 0.29 | 0.77 | 0.7 |
|  | **Leg2** | 0.32 | 0.38 | 0.34 | 0.68 | 0.66 |  | 0.29 | 0.3 | 0.29 | 0.76 | 0.71 |
|  | **Leg3** | 0.31 | 0.37 | 0.34 | 0.68 | 0.65 |  | 0.29 | 0.3 | 0.29 | 0.76 | 0.71 |
|  | **Leg4** | **0.31** | **0.37** | **0.34** | **0.68** | **0.65** |  | **0.29** | **0.3** | **0.29** | **0.75** | **0.7** |
| **Reduced model** | **Leg2r2** | 0.32 | 0.38 | 0.34 | 0.68 | 0.65 |  | 0.31 | 0.31 | 0.3 | 0.77 | 0.71 |
|  | **Leg3r2** | 0.32 | 0.38 | 0.34 | 0.69 | 0.65 |  | 0.31 | 0.32 | 0.3 | 0.77 | 0.71 |
|  | **Leg4r2** | 0.32 | 0.38 | 0.34 | 0.69 | 0.65 |  | 0.31 | 0.32 | 0.3 | 0.77 | 0.71 |
|  | **Leg3r3** | 0.32 | 0.38 | 0.34 | 0.68 | 0.66 |  | 0.3 | 0.3 | 0.29 | 0.76 | 0.71 |
|  | **Leg4r3** | 0.32 | 0.38 | 0.34 | 0.68 | 0.66 |  | 0.3 | 0.31 | 0.29 | 0.76 | 0.71 |
